# Construction and Validation of an Immune-Related Risk Score Model for Survival Prediction in Glioblastoma

**DOI:** 10.3389/fneur.2022.832944

**Published:** 2022-03-16

**Authors:** Wei Ren, Weifeng Jin, Zehua Liang

**Affiliations:** ^1^The First School of Clinical Medicine, Zhejiang Chinese Medical University, Hangzhou, China; ^2^School of Pharmaceutical Sciences, Zhejiang Chinese Medical University, Hangzhou, China; ^3^School of Humanities and Management, Zhejiang Chinese Medical University, Hangzhou, China

**Keywords:** glioblastoma, immune-related genes (IRGs), survival prognosis, risk score, tumor-infiltrating immune cells

## Abstract

**Background:**

As one of the most important brain tumors, glioblastoma (GBM) has a poor prognosis, especially in adults. Immune-related genes (IRGs) and immune cell infiltration are responsible for the pathogenesis of GBM. This study aimed to identify new tumor markers to predict the prognosis of patients with GBM.

**Methods:**

The Cancer Genome Atlas (TCGA) database and ImmPort database were used for model construction. The Wilcoxon rank-sum test was applied to identify the differentially expressed IRGs (DEIRGs) between the GBM and normal samples. Univariate Cox regression analysis and Kaplan–Meier analysis was performed to investigate the relationship between each DEIRG and overall survival. Next, multivariate Cox regression analysis was exploited to further explore the prognostic potential of DEIRGs. A risk-score model was constructed based on the above results. The area under the curve (AUC) values were calculated to assess the effect of the model prediction. Furthermore, the Chinese Glioma Genome Atlas (CGGA) dataset was used for model validation. STRING database and functional enrichment analysis were used for exploring the gene interactions and the underlying functions and pathways. The CIBERSORT algorithm was used for correlation analysis of the marker genes and the tumor-infiltrating immune cells.

**Results:**

There were 198 DEIRGs in GBM, including 153 upregulated genes and 45 downregulated genes. Seven marker genes (LYNX1, PRELID1P4, MMP9, TCF12, RGS14, RUNX1, and CCR2) were filtered out by sequential screening for DEIRGs. The regression coefficients (0.0410, 1.335, 0.005, −0.021, 0.123, 0.142, and −0.329) and expression data of the marker genes were used to construct the model. The AUC values for 1, 2, and 3 years were 0.744, 0.737, and 0.749 in the TCGA–GBM cohort and 0.612, 0.602, and 0.594 in the CGGA-GBM cohort, respectively, which indicated a high predictive power. The results of enrichment analysis revealed that these genes were enriched in the activation of T cell and cytokine receptor interaction pathways. The interaction network map demonstrated a close relationship between the marker genes MMP9 and CCR2. Infiltration analysis of the immune cells showed that dendritic cells (DCs) could identify GBM, while LYNX1, RUNX1, and CCR2 were significantly positively correlated with DCs expression.

**Conclusion:**

This study analyzed the expression of IRGs in GBM and identified seven marker genes for the construction of an immune-related risk score model. These marker genes were found to be associated with DCs and were enriched in similar immune response pathways. These findings are likely to provide new insights for the immunotherapy of patients with GBM.

## Introduction

Glioblastoma (GBM) is characterized by high mortality and accounts for 48.6% of primary malignant brain tumors (1). The incidence of GBM increases with age, with the highest incidence seen among people aged 75–84 years. The patients usually have an unfavorable prognosis, with a median survival time of 8 months [95% confidence interval (CI): 8–9] (1). Therefore, survival prediction is important and urgent for the treatment of patients with GBM.

In recent years, rapid developments in the field of bioinformatics have aided the exploration of molecular characteristics of cancer. Many new molecular markers and molecular characterization systems of GBM have emerged, which provide guidance for understanding the mechanism of progression and promote diagnosis and treatment (2). Studies related to mutations of isocitrate dehydrogenase and platelet-derived growth factor receptor (3), as well as promoter methylation of methylguanine methyltransferase show that considerable progress has been made in the genetic research of GBM biomarkers (4–6). However, favorable treatment options and effective prognostic markers remain insufficient. Therefore, clinicians and researchers need to identify novel GBM biomarkers to improve therapeutic accuracy.

Of late, advances in immunotherapy have proved that the immune microenvironment plays an important role in tumor biology and established the promising prospects of immunotherapy in the future (7, 8). The GBM microenvironment is usually immunosuppressive and consists of infiltrating immune cells, such as microglia, natural killer cells, and neutrophils. For example, the results of immunohistochemical analysis in literature (9) showed that more than 70% of the analyzed human glioma samples (*n* = 105) had significant neutrophil infiltration. And a higher degree of neutrophil infiltration was associated with higher malignant glioma grades (10, 11). However, the microenvironment lacks T cells and specific types of non-immune components, such as neurons, astrocytes, and tumor cells (7). Compared with most other types of tumors, the abundance of infiltrating T cells in glioblastoma is lower (8, 12). In addition, neurons, astrocytes and tumor cells are not the main types of infiltrating cells in GBM microenvironment, and they may play a role in GBM by direct synapses, pruning synapses and promoting synaptic formation (13, 14). Furthermore, which immune genes and immune cells are associated with the prognosis of GBM is yet to be elucidated. Thus, it is necessary to enhance our comprehension of the potential immunopathological mechanism of GBM progression.

In this study, RNA-Seq data were acquired from The Cancer Genome Atlas (TCGA) database and were matched with the list of immune-related genes (IRGs) in the ImmPort database. Next, the differentially expressed IRGs (DEIRGs) were identified, and a prognostic model was constructed *via* survival analysis and Cox proportional hazard regression analysis. The Chinese Glioma Genome Atlas (CGGA) data were used to validate the predictive value of the model. In addition, the biological functions and action pathways of seven marker genes in GBM were examined, and their correlation with 22 kinds of immune cells was analyzed. The results are expected to provide new insights to understand the pathogenesis of GBM and establish the strategies for immunotherapy.

## Methods

### Data Acquisition in GBM

The gene expression data and individual clinical information of GBM patients were derived from the TCGA and CGGA datasets. On excluding patient data that lacked information on the survival status and survival time, clinical information of 174 patients with TCGA and 657 patients with CGGA were obtained.

### Acquisition of IRGs

IRGs were exported from the ImmPort database, including IMMUNE_RESPONSE and IMMUNE_SYSTEM_PROCESS gene lists. A total of 332 genes were assessed in all (Supplementary Table 1).

### Variance Analysis

The data downloaded from the TCGA and CGGA databases based on log2 | FC | were first normalized. Then, the TCGA data were used to construct the model, and CGGA data were employed to test the predictive performance of the model in populations from different sources. Then, 56,763 expressed genes from 174 patients in TCGA were intersected with the list of immune-related genes, and 879 genes with the expression data were acquired. The “limma” package of R software was utilized to analyze the expression differences in the immune-related genes in TCGA-GBM patients, and the DEIRGs were screened out [the screening conditions were log2 | FC | > 2 and False Discovery Rate (FDR) < 0.05]. The “ggplot2” and “pheatmap” packages of the R software were used to visualize the final results.

### Screening and Identification of Marker Genes

The gene expression data of DEIRGs were integrated with the clinical data of TCGA-GBM patients including the survival status and the survival time data. The “survival” package of R software was employed for univariate Cox proportional hazard regression analyses and Kaplan–Meier analysis. *P* < 0.05 in both cases was considered to indicate the statistical difference. Then, the “survival” package of R software was employed for multivariate Cox proportional hazard regression analysis to further explore the prognostic potential of the obtained genes. The stepwise forward regression method was employed for multivariate Cox regression analysis, and the Akaike information criterion (AIC) was applied to avoid over-fitting. The genes with the largest likelihood ratios and the lowest AIC values were selected as the marker genes.

### Construction of Prognostic Model Based on Marker Genes

By combining the regression coefficient results of multivariate Cox regression analysis with the expression data of each marker gene, we established a risk score predictive model using the following calculation formula:


$$\begin{array}{l} Risk\;Score=(\beta_{1}\times gene_{1}expression)+(\beta_{2}\times gene_{2}expression)\\ \;+\cdots +(\beta_{n}\times gene_{n}expression) \end{array}$$

Where β corresponds to the regression coefficient.

### Evaluation and Validation of the Prognostic Model

According to the abovementioned model, we obtained the risk score of each patient and the grouping results of GBM patients. The survival curves were created using the Kaplan–Meier (KM) method, and the logarithmic rank test was employed to evaluate the different survival rates between the two groups. *P* < 0.05 was considered to indicate statistical significance. The “survivalROC” package of R software was used to draw the time-dependent receiver operating characteristic (ROC) curves, while the area under the curve (AUC) was applied to assess the specificity and sensitivity of the model. One and two years were defined as the time nodes. Survival analysis and AUC values were performed on CGGA data to validate the prognostic prediction performance of the prognostic model in Chinese GBM patients.

### Functional Enrichment Analyses

Gene Ontology (GO) analysis was applied to identify the common functions of DEIRGs. The Kyoto Encyclopedia of Genes and Genomes (KEGG) analysis was employed to identify the significant pathways for gene enrichment. The “clusterProfiler” package of R software was employed to display the enrichment results, and the significance threshold was set to 0.05. The STRING website was used to visualize the interactions between genes.

### Correlation Analysis of Immune Cell Infiltration

The CIBERSORT online site was referred for analyzing the differences in the infiltrate abundance of 22 immune cells between tumor samples and normal samples (15). Data from the Tumor IMmune Estimation Resource (TIMER) 2.0 database were used to analyze the relationship between marker genes and 22 types of immune cells in GBM patients (16). Data from the TIMER database were used to demonstrate the differential functions of individual immune cells between the tumor and normal samples (8, 17).

### Statistical Analyses

The ROC curves were employed to determine the diagnostic differentiation of the DEIRGs. KM analysis was performed to estimate the survival time of the two subgroups, while a logarithmic rank test was employed to determine the difference in prognosis (18). Cox proportional hazard regression analysis was applied to distinguish significant differences (18). All statistical calculation processes were conducted in the statistical software environment R version 4.1.0 or Microsoft Excel 2019. All *P-*values were two-tailed. The specific *P*-values and the other statistical methods are described throughout the study.

## Results

### Screening and Identification of DEIRGs

**Figure 1:**
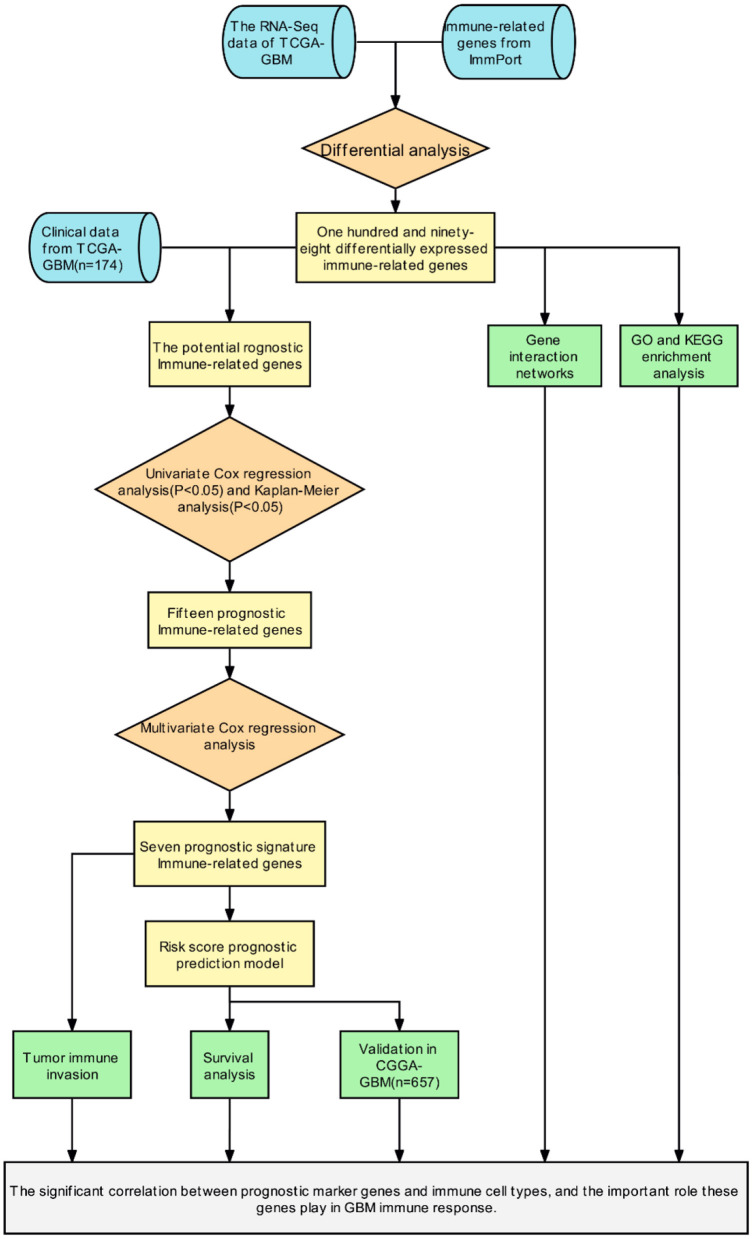
The flow chart of the study protocol.

**Figure 2:**
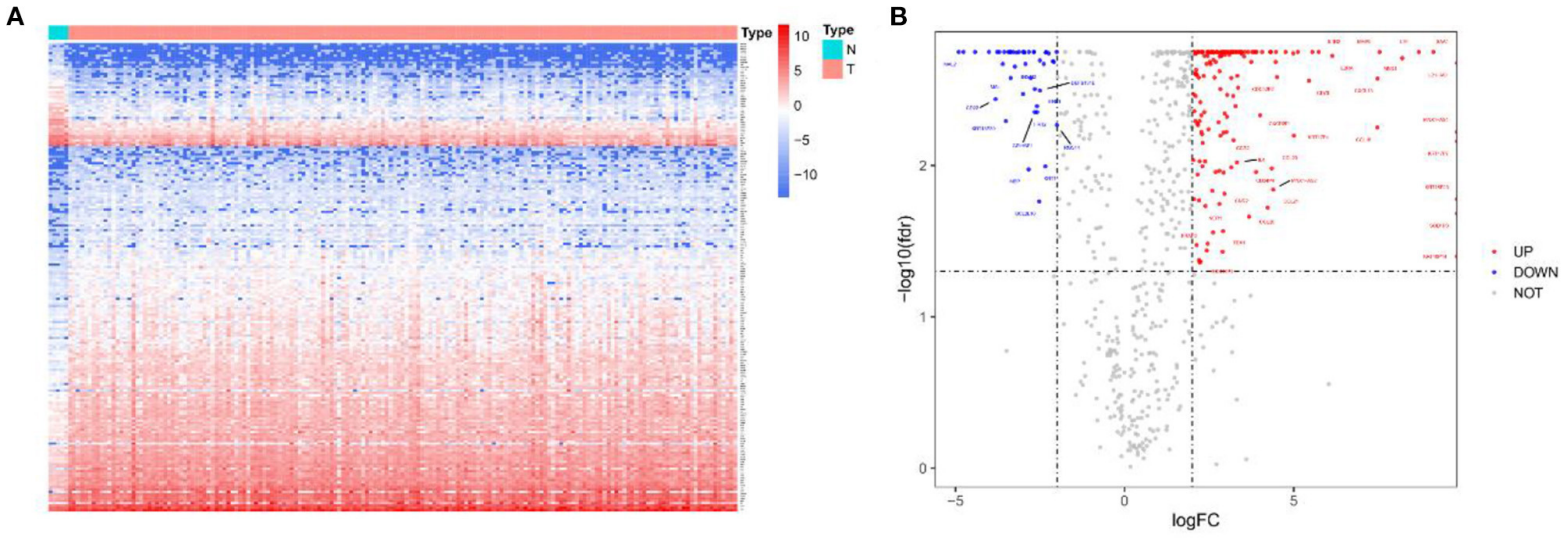
Heatmap and volcano plot of 198 DEIRGs in TCGA-GBM patients. **(A)** Heatmap of 198 DEIRGs in TCGA-GBM patients. Blue and red indicate the genes of a lower and higher expression. **(B)** Volcano plot of 198 DEIRGs in TCGA-GBM patients. Blue and red, respectively, represent the significantly downregulated and upregulated genes. The names of genes in the dense regions are hidden.

Initially, a flow chart was drawn to depict the analysis process (Figure 1). The TCGA-GBM data included 169 tumor samples and 5 normal samples. Upon comparing the expression abundance of IRGs between the tumor and normal samples, 198 DEIRGs satisfying both log2 | FC | > 2 and FDR < 0.05 were screened. The heat map revealed that 153 genes were upregulated and 45 genes were downregulated in the tumor samples (Supplementary Table 2). The volcano map signified that the log2 | FC | and –log10 (FDR) of RNA-Seq in the TCGA dataset. The genes with FDR < 0.05 and log2 FC > 2 (< -2) were marked with red (blue) dots. Figure 2 presents the results of gene screening.

### Establishment of Immune-Related Risk Score

**Figure 3:**
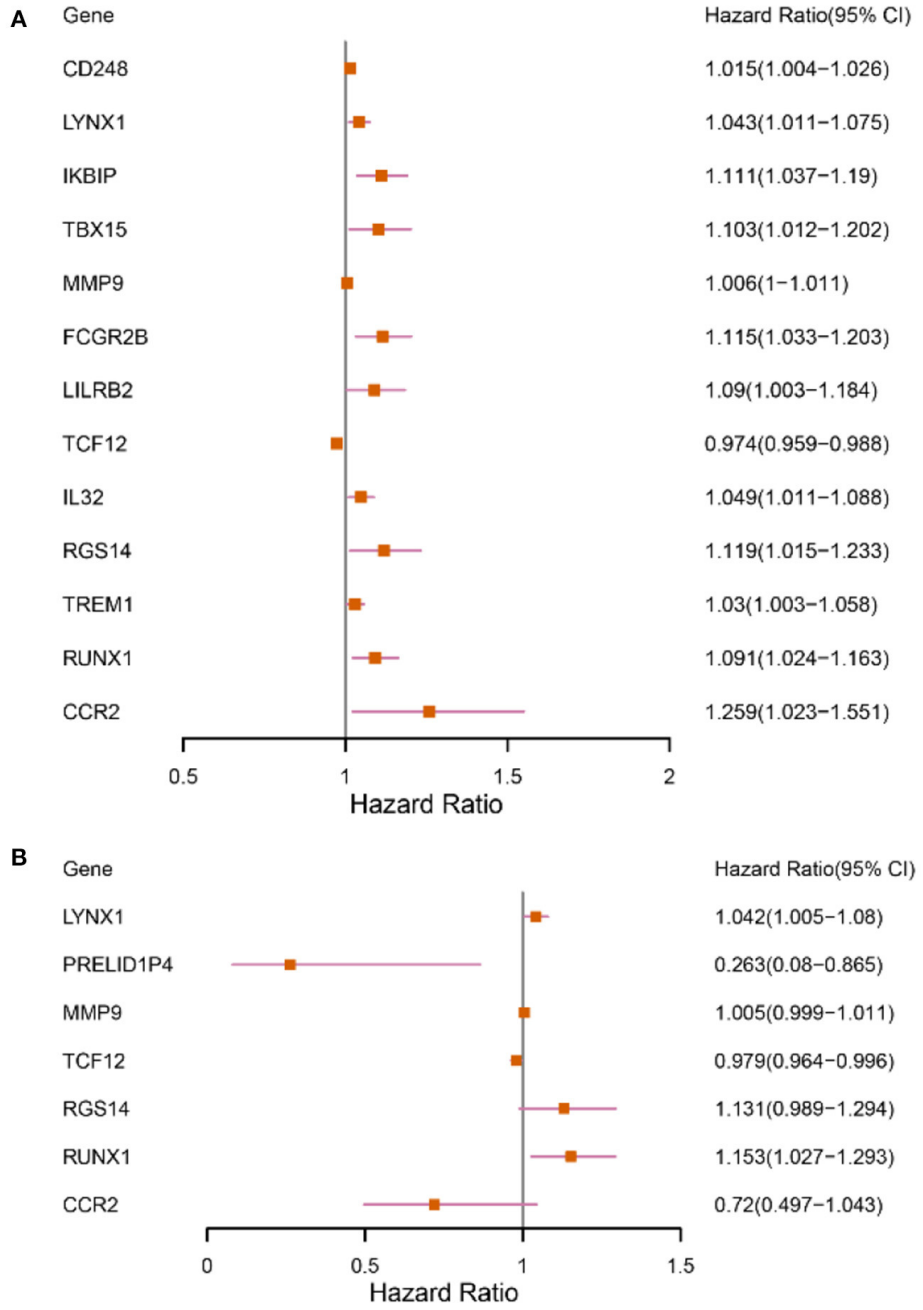
Forest plots depicting the results of univariate and multivariate Cox analyses. **(A)** The results of 13 DEIRGs were screened out by the univariate Cox analysis (We removed two of the genes with a large confidence interval). **(B)** The results of 7 prognostic DEIRGs were screened out by following multivariate Cox analyses.

The patients enrolled in the study were divided into the high- and the low-expression groups according to the median single-gene expression level. The single-gene KM curves showed that the hazard ratios (HRs) of the two groups remained the same throughout the study. Next, the DEIRGs were included in the univariate Cox regression analysis and the KM analysis assessed by the logarithmic rank test. Only DEIRGs with *P* < 0.05 in both cases were considered as candidate overall survival-related (OS-related) genes. By combining these two analyses, 15 genes were obtained (Table 1; Figure 3A). Single-gene KM curves of these 15 genes are shown in Supplementary Figure 1. To improve the independent forecasting ability of the model, these genes were included in the subsequent multivariate Cox regression analysis. Seven genes (LYNX1, PRELID1P4, MMP9, TCF12, RGS14, RUNX1, and CCR2) were obtained and were integrated into the prognosis model as marker genes (Table 2; Figure 3B).

**Table 1:** The results of the univariate Cox regression analysis.

**Gene**	**HR**	**95% CI**	**Cox *P*-value**	**Log-rank *P-*value**
CD248	1.015	1.004–1.026	0.008	0.016
LYNX1	1.043	1.011–1.075	0.007	0.030
PRELID1P4	0.254	0.079–0.815	0.021	0.043
IKBIP	1.111	1.037–1.190	0.003	0.019
RGS17P1	16.263	1.865–141.848	0.012	0.002
TBX15	1.103	1.012–1.202	0.025	0.040
MMP9	1.006	1.000–1.011	0.047	0.049
FCGR2B	1.115	1.033–1.203	0.005	0.021
LILRB2	1.090	1.003–1.184	0.041	0.027
TCF12	0.974	0.959–0.988	<0.001	0.003
IL32	1.049	1.011–1.088	0.011	0.042
RGS14	1.119	1.015–1.233	0.024	0.010
TREM1	1.030	1.003–1.058	0.027	0.006
RUNX1	1.091	1.024–1.163	0.007	0.027
CCR2	1.259	1.023–1.551	0.030	0.032

**Table 2:** The results of the multivariate Cox regression analyses.

**Gene**	**Coefficient**	**HR**	**95% CI**	**Cox *P*-value**
LYNX1	0.041	1.042	1.005–1.080	0.025
PRELID1P4	−1.335	0.263	0.080–0.865	0.028
MMP9	0.005	1.005	0.999–1.011	0.099
TCF12	−0.021	0.979	0.964–0.996	0.012
RGS14	0.123	1.131	0.989–1.294	0.072
RUNX1	0.142	1.153	1.027–1.293	0.016
CCR2	−0.329	0.720	0.497–1.043	0.083

Among the seven marker genes used to calculate the risk score, LYNX1, MMP9, RGS14, and RUNX1 were considered as the prognostic pathogenic genes, with HR > 1, while PRELID1P4, TCF12, and CCR2 were considered as the prognostic protection genes, with HR <1. The regression coefficients and gene expression data of the marker genes were used to establish a prognostic model. The following formula provides a method for calculating the risk score:


$$\begin{array}{l} Risk\;Score=0.041\times LYXN1-1.335\times PRELID1P4+0.005\\ \;\times MMP9-0.021\times TCF12+0.123\times RGS14\\ \;+0.142\times RUNX1-0.329\times CCR2 \end{array}$$

Based on the score formula, the risk score of each GBM was obtained. Next, the patients were divided into the high- and low-risk groups by taking the median value as the critical value.

### Accuracy Assessment and Validation of the Prognostic Model

**Figure 4:**
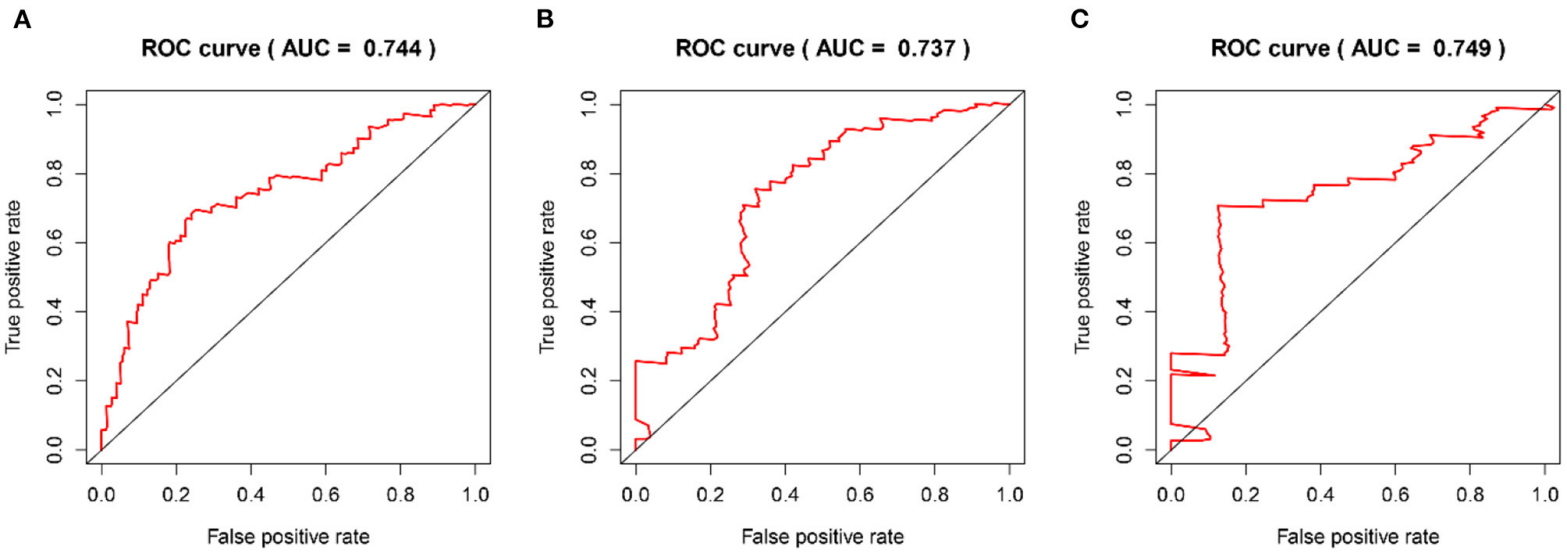
The time-dependent ROC curves in the TCGA training cohort. **(A–C)** ROC curves to evaluate the predictive accuracy of the model for OS at 1-, 2-, and 3-years in the TCGA training cohort.

**Figure 5:**
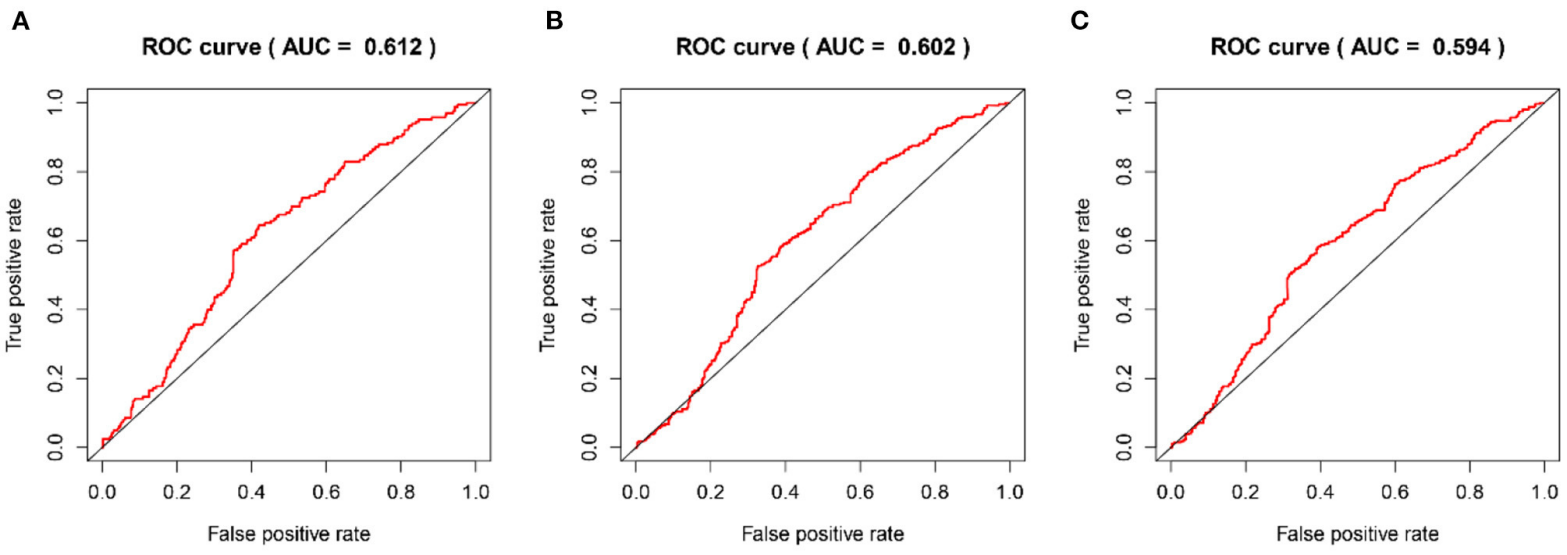
The time-dependent ROC curves in the CGGA validation cohort. **(A–C)** ROC curves to validate the predictive accuracy of the model for OS at 1-, 2-, and 3-years in the CGGA validation cohort.

The time-dependent ROC curves were employed to assess the predictive effect of the prognostic model. The AUC values of the model were 0.744 at 1 year, 0.737 at 2 years, and 0.749 at 3 years in the TCGA cohort (Figure 4) and 0.612 at 1 year, 0.602 at 2 years, and 0.594 at 3 years in the CGGA cohort (Figure 5).

**Figure 6:**
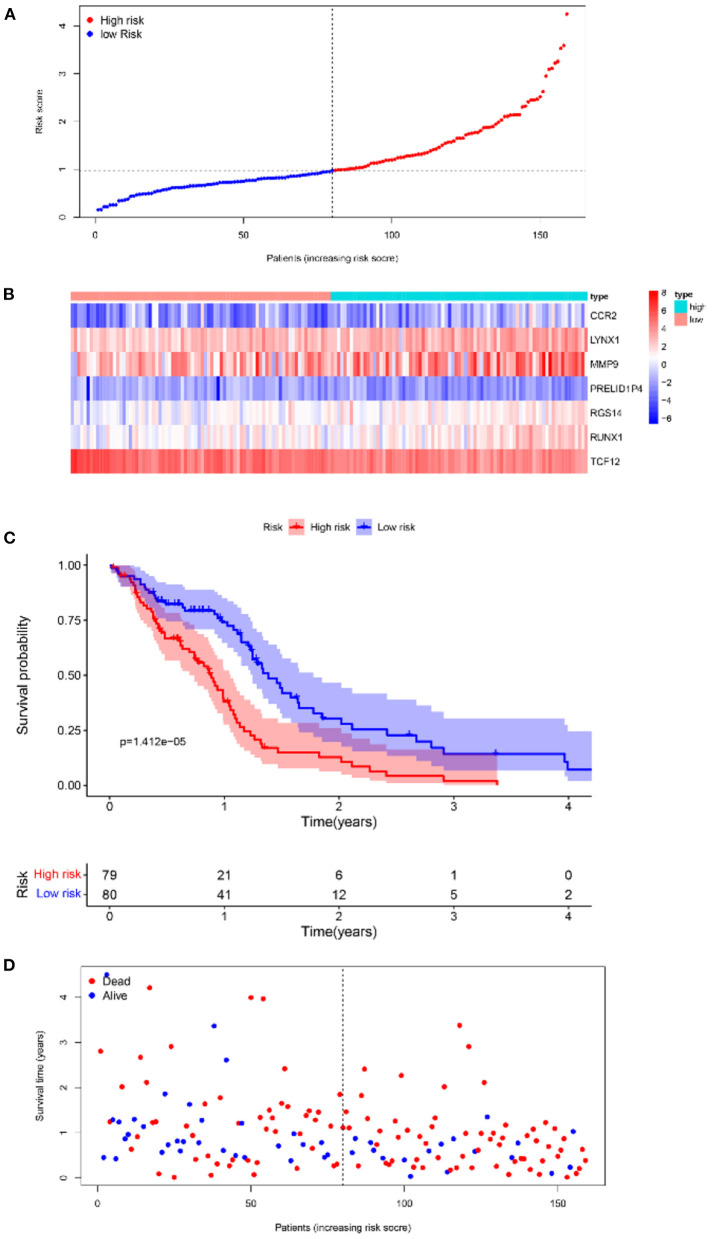
Survival analyses of the prognostic model in the TCGA-GBM cohort. **(A)** Risk score scatter map. **(B)** Heat map of differential expression of 7 marker genes. **(C)** The KM curves of OS in the high- and low-risk groups. **(D)** Survival status scatter map.

**Figure 7:**
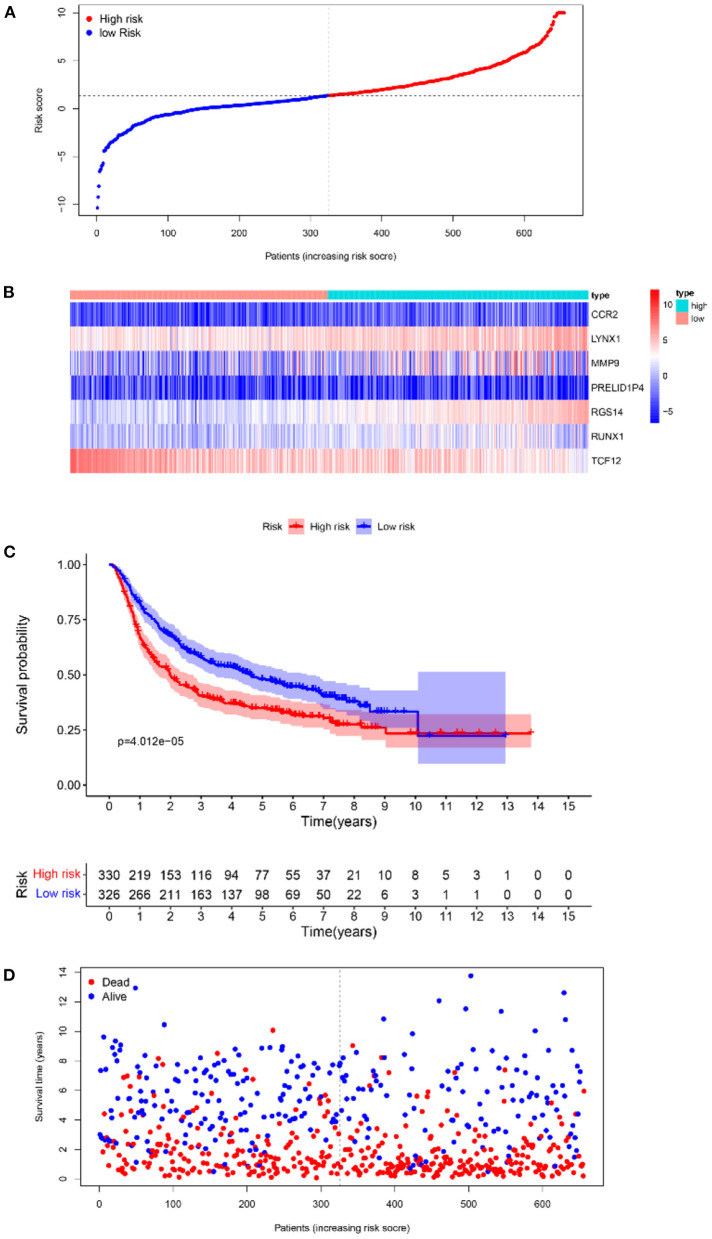
The results of survival analysis of the prognostic model in the CGGA-GBM cohort. **(A)** Risk score scatter map. **(B)** Heat map of the differential expression level of 7 marker genes. **(C)** KM curves of OS in the 2 risk subgroups. **(D)** Survival status scatter map.

Furthermore, survival analysis was performed in the model building and validation cohorts. Figure 6A shows the grouping results of all the patients with GBM in TCGA. The heatmap and the scatter map alluded that certain differences existed in the expression of the marker genes and OS (Figures 6B,D). These results were validated in the CGGA cohort (Figures 7A,B,D). The KM curves demonstrated that the low-risk group had a significantly better prognosis than the high-risk group, with a *P* < 0.05 (Figure 6C). Similar results were obtained in the CGGA validation cohort too, with *P* < 0.05 (Figure 7C). These findings suggest that the model has a good distinguishing ability and can be used as a prognostic biomarker in Chinese patients with GBM. In the training cohort and the validation cohort, the model in the first 3 years was better in distinguishing between high-risk and low-risk patients. In addition, we analyzed the effect of risk score on 15-year prognosis in the validation cohort, which shows that the model has a certain popularizing significance. However, in GBM cases, the survival time of more than 10 years is <1%, and long-term survivors are a very rare group (19). Shorter progression-free interval, larger diagnostic age, and surgical resection may be associated with a sudden decline in the 10-year survival probability of GBM patients (19).

### Enrichment Analysis

**Figure 8:**
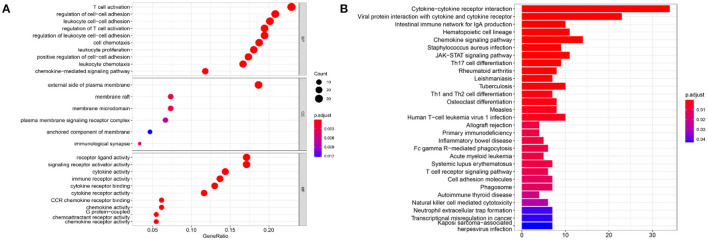
The results of functional enrichment analysis of 198 DEIRGs. **(A)** The results of GO analysis demonstrated the most-enriched biological functions. **(B)** The results of KEGG analysis show the most enriched biological processes. The X-axis represents the enrichment fraction, while the Y-axis represents the GO term and the KEGG pathway. The color indicates the *P*-value, while the circle size of the bubble chart represents the gene counts.

To infer the potential functions of DEIRGs in the carcinogenesis and development of GBM, the enrichment of GO terms and KEGG pathways of 198 DEIRGs were analyzed. GO enrichment analysis revealed 637 significantly enriched items. Supplementary Table 3 shows 26 GO items with the highest gene counts. T cell activation was found to be the most enriched in 584 items of biological processes, the outer surface of the plasma membrane was the most enriched in 6 items of cellular components, and receptor-ligand activity and signal receptor activator activity were the most enriched in 47 items of molecular function (Figure 8A). KEGG pathway enrichment analysis implied that the most significant pathway was the cytokine receptor interaction pathway (Figure 8B). Therefore, the DEIRGs mainly affect the internal functions of the immune cells, which may alter the immune response activity of the cells and affect the results of immunotherapy in the patients.

**Figure 9:**
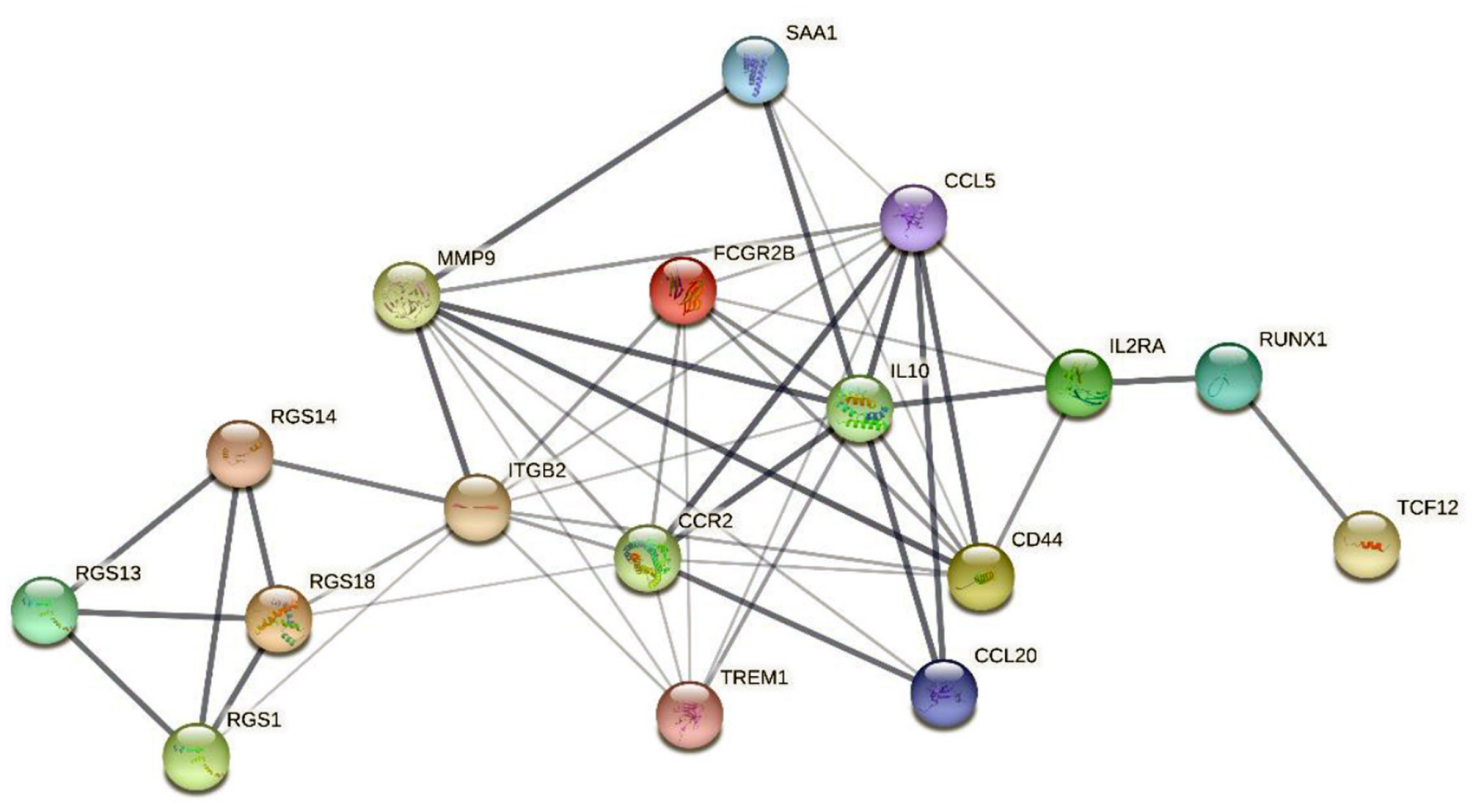
The interaction network map of 5 marker genes. The network nodes represent the protein types, the edges represent protein–protein associations, and the line thicknesses represent the strength of the data supporting the interaction between the genes.

In addition, gene–protein interaction was explored on the STRING website (Supplementary Table 4). The website contains comprehensive known and predicted protein–gene interactions (20, 21). STRING was employed to explore the interaction between the marker genes and the realization mechanism of the regulatory functions. Figure 9 shows the interactions of the five marker genes.

The interaction network map demonstrated that MMP9 and CCR2 were enriched in the central region of the network and exhibited strong interactions with several DEIRGs. In addition, RGS14 was linked to the central genes *via* interactions with ITGB2 and the RGS gene family. RUNX1 and TCF12 were connected to the central genes chiefly *via* interactions with the associated genes IL2RA and IL10.

### Analysis of Tumor Immune Infiltration

**Figure 10:**
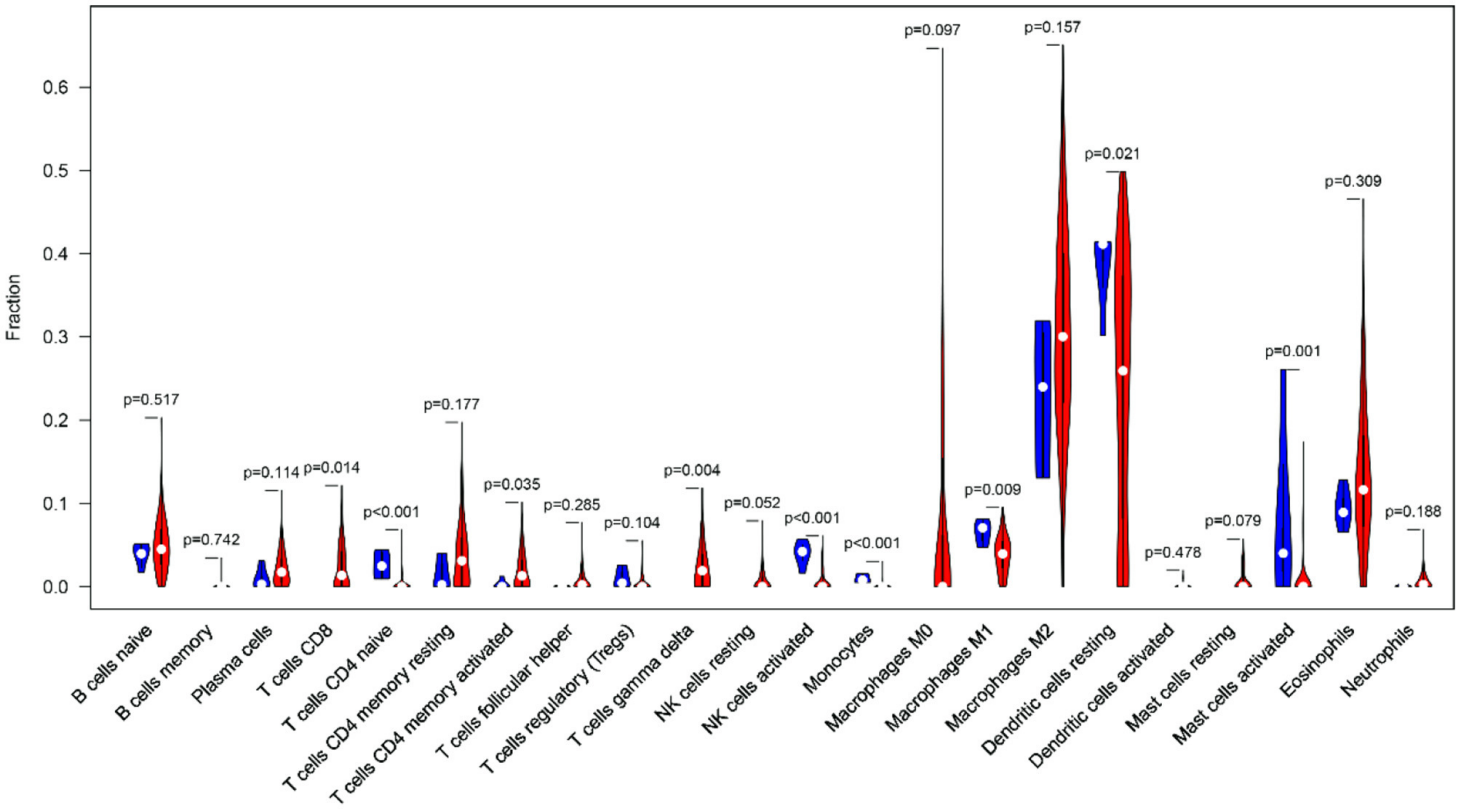
The violin plot shows the infiltration levels of 22 types of immune cells (blue and red represent normal and GBM samples, respectively).

Clinical studies on immunotherapy have confirmed that tumor-infiltrating immune cells can serve as a reference for the prognosis and immunotherapy of some solid tumors (22, 23). The tumor microenvironment plays a key role in tumor pathogenesis (24, 25). The CIBERSORT algorithm was applied to analyze the differences in the 22 immune cells between the tumor samples and the normal samples (Figure 10). The expression data of the 879 IRGs in the patients with GBM in TCGA were included, and the perturbation times were set to 1,000.

The violin plot indicated that the M2 macrophages and the resting dendritic cells (DCs) had high invasion scores in the tumor samples (infiltration fraction > 0.2). This finding had implications for the important roles of these two types of cells in the tumor microenvironment. In the GBM tissues, tumor-associated macrophages mainly originated from the peripheral blood monocytes, and M2 polarization was induced by ARS2/MAGL signals from the tumor stem cells (26, 27). DCs can not only induce tumor antigen specific immune response by recognizing and presenting immature antigens, but also directly or indirectly participate in the regulation of immune system (28). In addition, there was a significant infiltration-level difference in the DCs between the normal and GBM tissues (*P* < 0.05). The accumulation of eosinophils has been found in a variety of central nervous system diseases, but the involvement of eosinophils in the immune response in GBM has only been preliminarily recognized in some case studies (29). Eosinophils may regulate the function of other immune cells by producing cytokines and chemokines and activate infiltrating immune cells in the tumor microenvironment (30). This may provide an explanation for GBM identification and immunotherapy.

**Figure 11:**
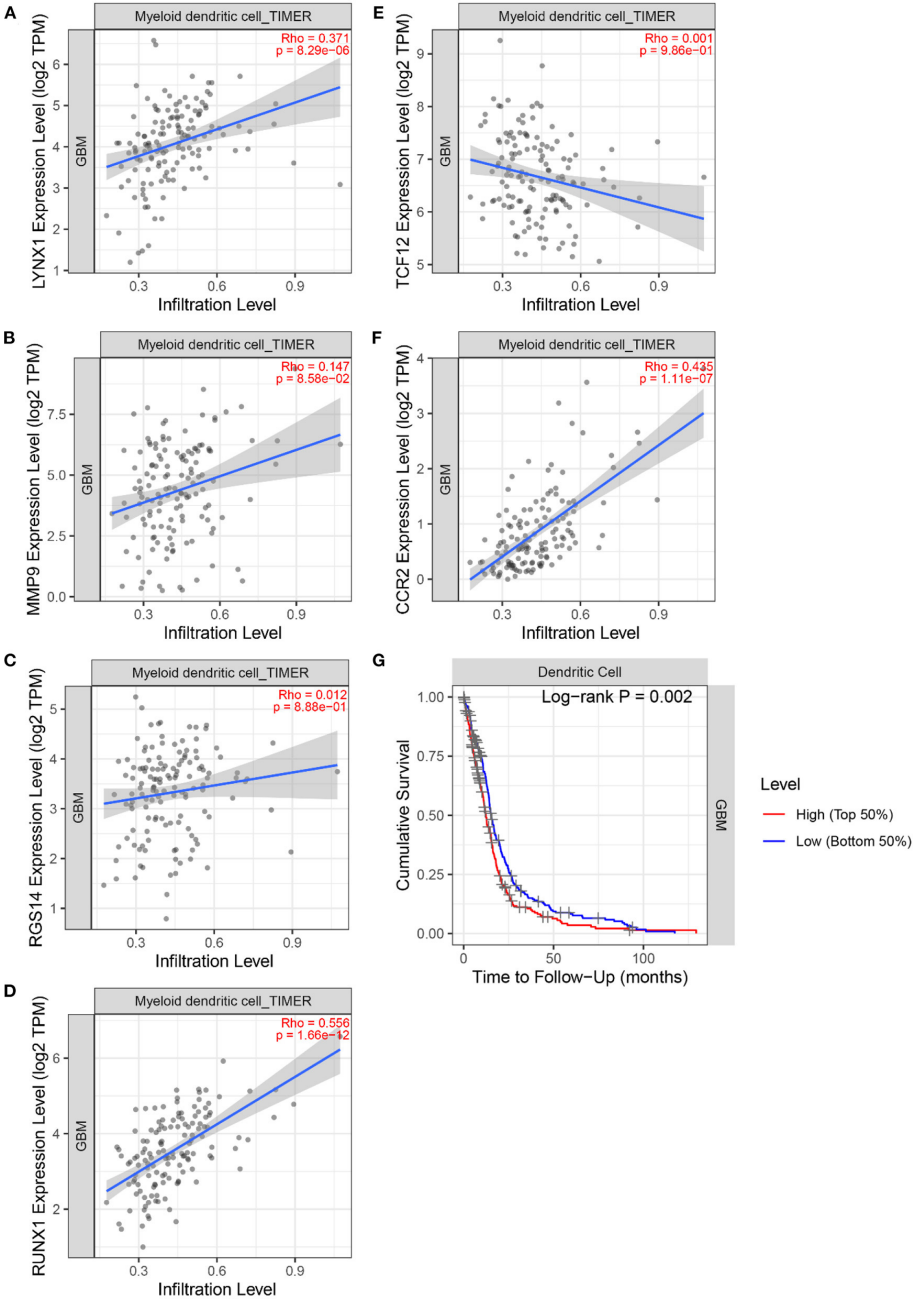
The correlation between 6 marker genes and DC. **(A)** LYNX1. **(B)** MMP9. **(C)** TCF12. **(D)** RGS14. **(E)** RUNX1. **(F)** CCR2. **(G)** Kaplan–Meier analysis of the DC infiltrating levels from the TCGA database.

Next, the TIMER 2.0 database was used to describe the correlation between the six marker genes and the DCs (Figures 11A–F). Furthermore, the TIMER database was used to generate KM curves for the DCs based on the TCGA-GBM gene expression data (Figure 11G).

Figure 11 demonstrates the correlation coefficients ρ and *P*-values between the expression levels of six marker genes and the infiltration abundance of DCs. All ρ > 0 indicated that the six marker genes were positively correlated with DCs. Moreover, the ρ > 0.3 and *P* < 0.05 of LYNX1, RUNX1, and CCR2 suggested their significant positive correlation with DCs. These three genes may affect the tumor immune microenvironment and regulate the growth and invasion rate of the tumor cells by affecting the invasion of the DCs. In particular, Dendritic Cell-Specific Intercellular adhesion molecule-3-Grabbing Non-integrin (DC-SIGN) is highly expressed on immature DCs (31). And the activation of DC-SIGN promotes the transcription of MMP9 (32). Literature (33) shows that human monocyte-derived DCs express a large amount of RGS14, and the expression level of RGS protein family can be changed by toll-like receptor signals. Literature (34) found that TNF- α induces DC-SIGN expression in renal tubular epithelial cells and may be regulated through MTOR-RUNX1 pathway. In Helicobacter pylori infection model, the loss of CCR2 signal leads to the defect of differentiation and maturation of DCs, which affects the immune response function of DCs (35). Furthermore, the logarithmic rank *P* < 0.05 of DC was obtained. DCs can recognize and present foreign antigens and regulate the growth of tumors in the central nervous system by increasing the activity of natural killer cells and natural killer T cells (36). However, a small amount of circulating DCs in the body may inhibit endogenous tumor immunity (36). Individualized DC vaccines based on tumor-associated antigens have been developed on this basis and have been proved to be safe and effective in some GBM treatments (37). This finding signifies that the DCs are an important type of tumor-infiltrating immune cells equipped with the ability to identify GBM tumors.

## Discussion

As one of the most invasive brain tumors, GBM is characterized by an unfavorable prognosis. Immunotherapy may prolong the survival time of the patients to some extent in the future. By analyzing the IRGs in the TCGA-GBM and ImmPort databases, 198 DEIRGs were identified. By employing several dimensionality reduction methods, such as difference analysis and Cox proportional hazard regression analysis, seven genes capable of predicting OS were screened, namely, LYNX1, PRELID1P4, MMP9, TCF12, RGS14, RUNX1, and CCR2. According to the Cox proportional hazard regression coefficients of the seven marker genes, a method for calculating the immune-related risk score was determined. On the basis of the sample coding rules of TCGA and CGGA databases (38, 39), patients with a risk score higher than the median value were considered to have a high risk for poor prognosis. The immune-related risk score can be used as a new prognostic predictor to explain the toxic role in immunobiology from cellular and molecular pathways (40). It may prevent the occurrence of immune-related adverse events by early identification of patients at risk of immunotherapy, so as to continue to maintain and give full play to the advantages of tumor immunotherapy (40). In addition, differential expression analysis of the marker genes revealed the significantly elevated expression of five genes (PRELID1P4, MMP9, TCF12, RUNX1, and CCR2) and the significantly lowered expression of two genes (LYNX1 and RGS14). Functional analysis showed that these genes were primarily involved in T cell activation and that they made a difference *via* cytokine receptor interaction pathways. In addition, the marker genes can be used as characteristic immune cell-specific genes, and they are also the expression products of some stromal cells in GBM microenvironment (8). This not only provides an explanation for the pattern of GBM-specific immune infiltration, but also opens a new window for the pathogenesis of immune infiltration in GBM.

The characteristics of the seven marker genes provide rich biological knowledge and potential therapeutic information about GBM. MMP9, one of the marker genes, is a oncogene of GBM (41). Studies have shown that the expression of MMP9 is upregulated in the GBM tissues and that it has the potential to induce the proliferation of GBM, thus worsening the prognosis of patients with GBM (41). In GBM patients, MMP9 is closely associated with hematopoietic progenitor cells, which may contribute to the development of specific therapies aimed at reducing HPC (42). Moreover, the association and interaction between heat shock protein 27 and MMP9 may contribute to the development of drugs that inhibit the infiltration and migration of GBM. This interaction also provides a new field of vision for the treatment of GBM (43). TCF12 has been proved to be an important target for miR-154 and can regulate the epithelial-mesenchymal transformation of GBM (44). P53/miR-154/TCF12 pathway may be involved in inhibiting the growth and invasion of GBM cells, so it can be used as a potential therapeutic target (44). Another marker gene, RGS14, is highly expressed in the caudate nucleus of the brain and thymus (45–47). Previous studies have shown that RGS14 targets the shuttle between centrosome and nucleoplasm and participates in the regulation of stress-induced cellular response (46). However, the role of RGS14 in the tumor tissues is yet to be elucidated. It was found that RGS14 had a low expression in GBM tissues, but it was potentially tumorigenic. More work is needed to discover the different roles of RGS14 in the GBM tissues and normal tissues. Another marker gene, RUNX1, is highly expressed in GBM, especially of the mesenchymal subtype (Mes). RUNX1 may promote the invasion of GBM cells by inducing TGF β signal transduction. The Mes GBM is widely seen in samples showing a high degree of necrosis. As an important transcriptional regulator of mesenchymal transformation, RUNX1 is also closely related to tissue necrosis (48, 49). This finding indicates that RUNX1 is a key signal node and that it might have a potential role in targeted therapy (50). Furthermore, CCL2 and its related receptor (CCR2) play an important role in brain tumors and are involved in regulating the migration of monocytes into the vascular endothelium. CCR2 inhibition can reduce tumor myeloid cells, suggesting that it may play a role in delaying the progression of gliomas (51–53).

The results of GO analysis showed that the most significant enrichment of biological process was T cell activation. This result suggests that immune-related genes may cause alterations in the morphology and maturity of T cells. Studies have revealed that PD-L1 present on the surface of GBM promotes the activation of PD-1 receptor in microglia, which inhibits the continuous proliferation of T cells and downregulates the cytotoxic activity of lymphocytes (54). Furthermore, regulation of leukocyte cell–cell adhesion was also highly enriched. For example, proteins regulated by IL-1 β may alter the frequency and speed of monocytes adhering to basal cells (55). The most significant enrichment of Cellular Component showed that the differential immune genes mainly performed biological functions on the extracellular side of the plasma membrane. The most significant enrichments of Molecular Function were the activities of receptor-ligand and signal receptor activator. For example, PD-1 ligand and PD-L1 receptor can negatively regulate T cell response and maintain homeostasis of the tumor microenvironment. Related studies have shown that PD-L1 can be used as a biomarker to assess the World Health Organization classification of GBM (54). For example, mutations or overexpression of epidermal growth factor receptors can activate the downstream signaling pathways, which may explain the pathogenesis of GBM (56, 57). On the other hand, the activity of the signal receptor activator may be correlated with the activation of the signaling pathway. CMTM6 is a key regulator of PD-L1, regulating T cell activity *in vivo* and *in vitro* (58). High levels of CMTM6 may inhibit the anti-tumor immune response of T cells in GBM, so the overexpression of CMTM6 is associated with poor prognosis and short overall survival of GBM patients (58). As for KEGG enrichment results, cytokine receptor interaction was the most important pathway. Moreover, other pathways, such as the interaction of viral proteins with cytokines and cytokine receptors and the JAK-STAT signaling pathways, were correlated with tumorigenesis (59, 60).

Furthermore, studies have shown that high immune infiltration in GBM predicts a poor outcome (61). Resting DC infiltration is a prominent feature of the GBM microenvironment in preclinical models and clinical samples. DC is an important immune cell in both innate and adaptive immune systems and has a complex array of functions and phenotypes in GBM microenvironment. Improvements in immunotherapy for DCs are in the experimental stage (62–64). In addition, the antitumor immune response of the DCs in the GBM tumor microenvironment may be realized by interaction with microglia, tumor cells, and T cells (62). Specifically, the combined application of CCR2 + HSCs and anti-PD-1 has been proven to significantly increase the median and long-term survival rates in the GBM model (65). The main mechanism of MMP9 in the brain is that it promotes enzyme activity and activates various cytokine- and chemokine-related immune/inflammatory reactions. Moreover, MM9 can promote leukocyte extravasation into the brain parenchyma and aid in destroying the blood–brain barrier. Its improper release may cause the development of brain tumors (66).

The TCGA-GBM-based risk score prognostic model was further validated in the Chinese samples, which indicated that our model has a certain degree of robustness. However, the results of this study are only based on bioinformatics and can only be used as a reference for preclinical research. In the future, more experiments are needed to prove it, such as flow cytometry and immunohistochemistry, to open up new ideas for the development of immunotherapy for GBM.

## Conclusion

In this study, an immune-related risk score was established based on TCGA-GBM and ImmPort databases, and the predictive effect was validated using the CGGA-GBM database. Based on the findings, it could be suggested that the seven-gene risk score can independently evaluate the survival of patients with GBM. In addition, this score may partly reflect the immune microenvironment of tumor infiltration *via* the association between marker genes and DCs. The score is also closely related to the response rate of immunotherapy, thus serving as an immunotherapy reference for patients with GBM.

## Data Availability Statement

The datasets presented in this study can be found in online repositories. The names of the repository/repositories and accession number(s) can be found in the article/Supplementary Material.

## Author Contributions

WR and ZL contributed to research concepts and design, while WR and WJ contributed to data collection and statistical analysis. All authors contributed to the critical revision of important intellectual content of the manuscript.

## Funding

This work was jointly supported by the National Natural Science Foundation of China (No. 81973560) and the Key Laboratory of TCM Encephalopathy of Zhejiang Province (Grant No. 2020E10012).

## Conflict of Interest

The authors declare that the research was conducted in the absence of any commercial or financial relationships that could be construed as a potential conflict of interest.

## Publisher's Note

All claims expressed in this article are solely those of the authors and do not necessarily represent those of their affiliated organizations, or those of the publisher, the editors and the reviewers. Any product that may be evaluated in this article, or claim that may be made by its manufacturer, is not guaranteed or endorsed by the publisher.
