# Supplementary material for: Construction and Validation of an Immune-Related Risk Score Model for Survival Prediction in Glioblastoma
Source: Front Neurol. 2022 Mar 16;13:832944. doi: 10.3389/fneur.2022.832944 (PMC8965766; doi:10.3389/fneur.2022.832944)
Supplement: Supplementary file 1 [file Table_1.DOCX]

Supplementary Material

# Supplementary Data

1.Data 1. Immune-related genes obtained from ImmPort database.

| Gene Code | Gene Name | Gene Code | Gene Name | Gene Code | Gene Name |
| --- | --- | --- | --- | --- | --- |
| 1 | ACIN1 | 112 | DEFB118 | 223 | MAFB |
| 2 | ACKR2 | 113 | DEFB127 | 224 | MAL |
| 3 | ACKR4 | 114 | DEFB4A | 225 | MALT1 |
| 4 | ACVR1B | 115 | DMBT1 | 226 | MAP3K7 |
| 5 | ACVR2A | 116 | DOCK2 | 227 | MAP4K1 |
| 6 | ADGRE5 | 117 | DPP4 | 228 | MAP4K2 |
| 7 | AIM2 | 118 | DPP8 | 229 | MBL2 |
| 8 | AIMP1 | 119 | DYRK3 | 230 | MBP |
| 9 | AKT1 | 120 | EBI3 | 231 | MIA3 |
| 10 | ALAS2 | 121 | ELF4 | 232 | MLF1 |
| 11 | ANXA11 | 122 | ELP1 | 233 | MMP9 |
| 12 | APLN | 123 | ERAP2 | 234 | MNX1 |
| 13 | APOA1 | 124 | EREG | 235 | MR1 |
| 14 | APOA2 | 125 | ETS1 | 236 | MS4A1 |
| 15 | APOA4 | 126 | FCAR | 237 | MS4A2 |
| 16 | APOBEC3F | 127 | FCGR1A | 238 | MYH9 |
| 17 | APOBEC3G | 128 | FCGR2B | 239 | NCF4 |
| 18 | AQP9 | 129 | FCGR3A | 240 | NCK1 |
| 19 | ARHGDIB | 130 | FCGR3B | 241 | NCK2 |
| 20 | ATP6V0A2 | 131 | FCGRT | 242 | NCOA6 |
| 21 | AZU1 | 132 | FCN1 | 243 | NCR1 |
| 22 | BCAR1 | 133 | FCN2 | 244 | NFAM1 |
| 23 | BCL10 | 134 | FOXO3 | 245 | NFIL3 |
| 24 | BCL2 | 135 | FOXP3 | 246 | NHEJ1 |
| 25 | BLNK | 136 | FTH1 | 247 | NLRC3 |
| 26 | BNIP3 | 137 | FYB1 | 248 | NOTCH2 |
| 27 | BNIP3L | 138 | FYN | 249 | NOTCH4 |
| 28 | BST1 | 139 | GBP2 | 250 | OPRD1 |
| 29 | BST2 | 140 | GEM | 251 | OPRK1 |
| 30 | C1QBP | 141 | GLMN | 252 | PAX5 |
| 31 | C2 | 142 | GPI | 253 | PDCD1 |
| 32 | C5AR1 | 143 | GPR183 | 254 | PF4 |
| 33 | CADM1 | 144 | GPR65 | 255 | POU2AF1 |
| 34 | CALCA | 145 | GTPBP1 | 256 | POU2F2 |
| 35 | CARTPT | 146 | GZMA | 257 | PRELID1 |
| 36 | CCL18 | 147 | HAMP | 258 | PREX1 |
| 37 | CCL19 | 148 | HCLS1 | 259 | PRG3 |
| 38 | CCL2 | 149 | HDAC4 | 260 | PRKRA |
| 39 | CCL20 | 150 | HDAC5 | 261 | PRL |
| 40 | CCL21 | 151 | HDAC7 | 262 | PSMB10 |
| 41 | CCL22 | 152 | HDAC9 | 263 | PTAFR |
| 42 | CCL23 | 153 | HELLS | 264 | PTGDR2 |
| 43 | CCL24 | 154 | HLA-DRB3 | 265 | PTGER4 |
| 44 | CCL25 | 155 | HRH2 | 266 | PTPRC |
| 45 | CCL26 | 156 | ICOSLG | 267 | PYDC1 |
| 46 | CCL27 | 157 | IFI16 | 268 | RAB3D |
| 47 | CCL4 | 158 | IFI6 | 269 | RAG1 |
| 48 | CCL5 | 159 | IFITM2 | 270 | RASGRP4 |
| 49 | CCR1 | 160 | IFITM3 | 271 | RFX1 |
| 50 | CCR2 | 161 | IFNK | 272 | RGS1 |
| 51 | CCR4 | 162 | IFNL1 | 273 | RPS19 |
| 52 | CCR5 | 163 | IFNLR1 | 274 | RSAD2 |
| 53 | CCR6 | 164 | IGSF6 | 275 | RUNX1 |
| 54 | CCR8 | 165 | IK | 276 | S1PR4 |
| 55 | CCR9 | 166 | IKBKG | 277 | SAA1 |
| 56 | CD164 | 167 | IL10 | 278 | SART1 |
| 57 | CD1D | 168 | IL10RB | 279 | SCG2 |
| 58 | CD2 | 169 | IL12A | 280 | SCIN |
| 59 | CD22 | 170 | IL12B | 281 | SECTM1 |
| 60 | CD24 | 171 | IL15 | 282 | SEMA3C |
| 61 | CD274 | 172 | IL16 | 283 | SEMA4D |
| 62 | CD276 | 173 | IL17A | 284 | SEMA7A |
| 63 | CD28 | 174 | IL17B | 285 | SFTPD |
| 64 | CD34 | 175 | IL18 | 286 | SIRPG |
| 65 | CD3D | 176 | IL18BP | 287 | SIT1 |
| 66 | CD3E | 177 | IL1R2 | 288 | SKAP1 |
| 67 | CD4 | 178 | IL2 | 289 | SLA2 |
| 68 | CD40LG | 179 | IL21 | 290 | SNRK |
| 69 | CD47 | 180 | IL27 | 291 | SOCS5 |
| 70 | CD7 | 181 | IL27RA | 292 | SOD1 |
| 71 | CD74 | 182 | IL2RA | 293 | SP2 |
| 72 | CD79A | 183 | IL2RG | 294 | SPACA3 |
| 73 | CD79B | 184 | IL31RA | 295 | SPI1 |
| 74 | CD83 | 185 | IL32 | 296 | SPINK5 |
| 75 | CD86 | 186 | IL4 | 297 | ST6GAL1 |
| 76 | CD96 | 187 | IL4R | 298 | SYK |
| 77 | CDC42 | 188 | IL6 | 299 | TAPBP |
| 78 | CDK6 | 189 | IL6R | 300 | TARBP2 |
| 79 | CEACAM8 | 190 | IL6ST | 301 | TAZ |
| 80 | CEBPB | 191 | IL7 | 302 | TBX1 |
| 81 | CEBPG | 192 | IL7R | 303 | TCF12 |
| 82 | CFHR1 | 193 | INHA | 304 | TCF7 |
| 83 | CHST4 | 194 | INHBA | 305 | TENM1 |
| 84 | CHUK | 195 | INS | 306 | TGFB1 |
| 85 | CIITA | 196 | IRAG2 | 307 | TGFB2 |
| 86 | CKLF | 197 | IRF8 | 308 | THY1 |
| 87 | CLEC7A | 198 | ITGB2 | 309 | TLR4 |
| 88 | CMKLR1 | 199 | JAG2 | 310 | TLR7 |
| 89 | CNIH1 | 200 | KAT6A | 311 | TLR8 |
| 90 | CNR2 | 201 | KAT8 | 312 | TNFAIP1 |
| 91 | COLEC12 | 202 | KIR2DL1 | 313 | TNFRSF14 |
| 92 | CRHR1 | 203 | KIR2DL3 | 314 | TNFRSF4 |
| 93 | CRTAM | 204 | KIRREL3 | 315 | TNFSF13 |
| 94 | CSF1 | 205 | KMT2A | 316 | TPD52 |
| 95 | CST7 | 206 | KRT1 | 317 | TRAF2 |
| 96 | CTLA4 | 207 | LAT | 318 | TRAF6 |
| 97 | CTSC | 208 | LAT2 | 319 | TRAT1 |
| 98 | CTSE | 209 | LAX1 | 320 | TREM1 |
| 99 | CTSG | 210 | LCK | 321 | TREM2 |
| 100 | CTSS | 211 | LCP2 | 322 | TRIM22 |
| 101 | CTSW | 212 | LDB1 | 323 | UBE2N |
| 102 | CX3CL1 | 213 | LIG1 | 324 | VIPR1 |
| 103 | CXCL12 | 214 | LIG3 | 325 | VTN |
| 104 | CXCL13 | 215 | LILRB2 | 326 | WAS |
| 105 | CXCL8 | 216 | LST1 | 327 | XBP1 |
| 106 | CXCR2 | 217 | LTB4R | 328 | YTHDF2 |
| 107 | CXCR4 | 218 | LTF | 329 | ZAP70 |
| 108 | DCSTAMP | 219 | LY75 | 330 | ZBTB16 |
| 109 | DEFA1 | 220 | LY86 | 331 | ZEB1 |
| 110 | DEFB1 | 221 | LYN | 332 | ZNF675 |
| 111 | DEFB103A | 222 | MADCAM1 |  |  |

# Supplementary Figures and Tables

## Supplementary Figures


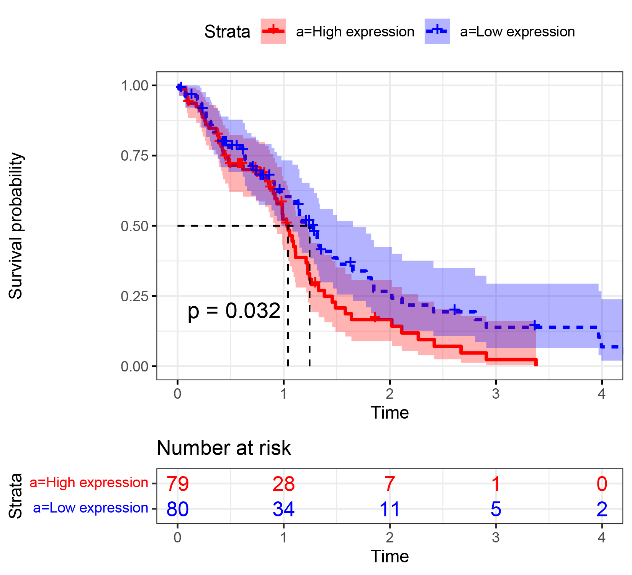

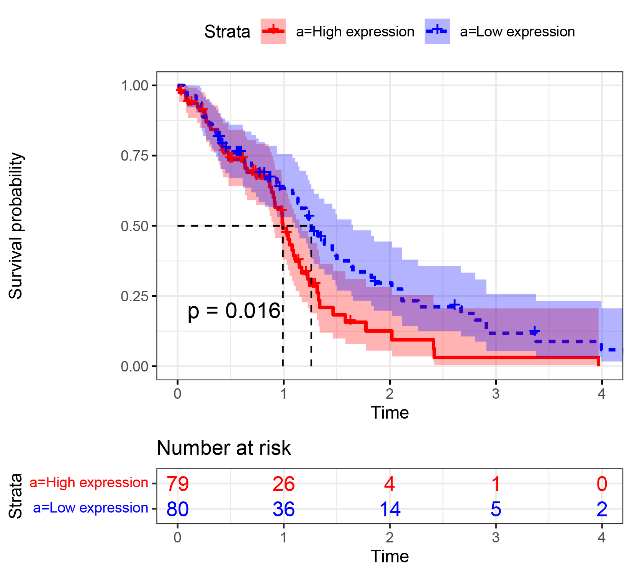


(A) (B)


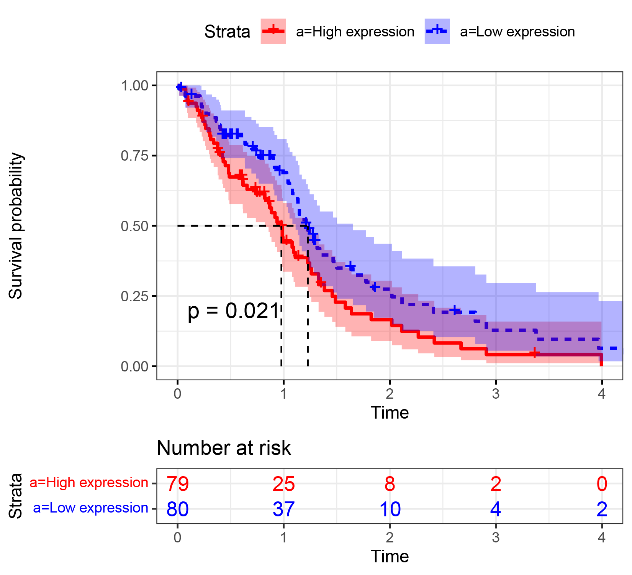

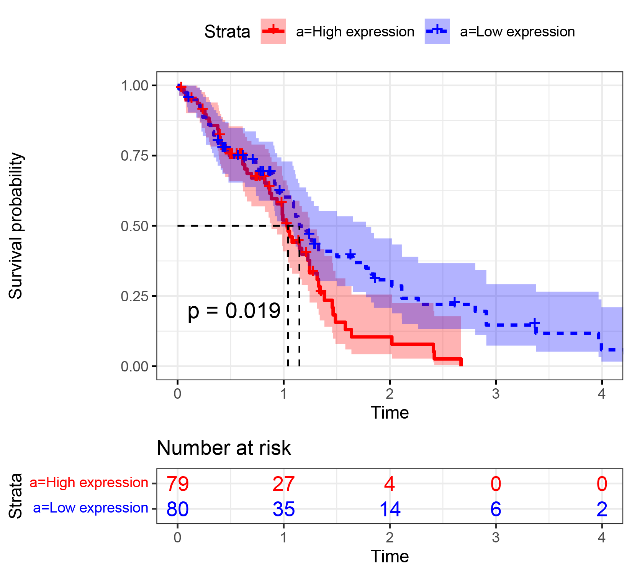


(C) (D)


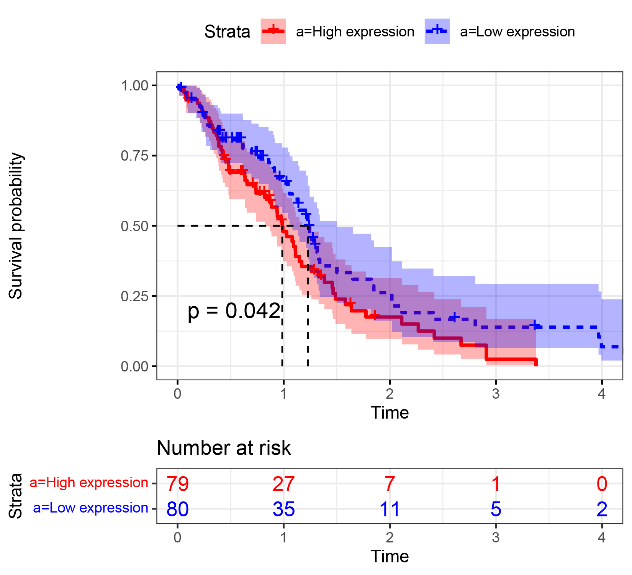

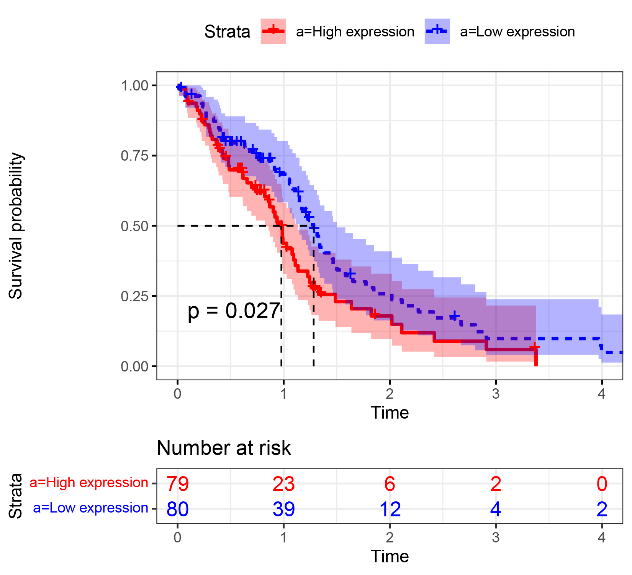


(E) (F)


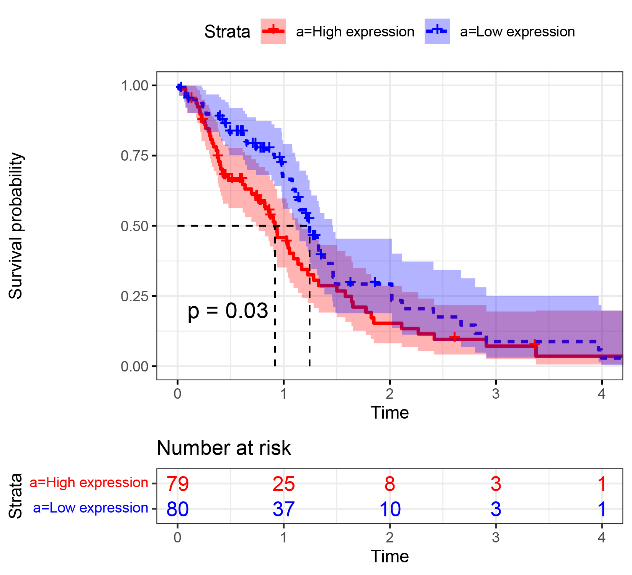

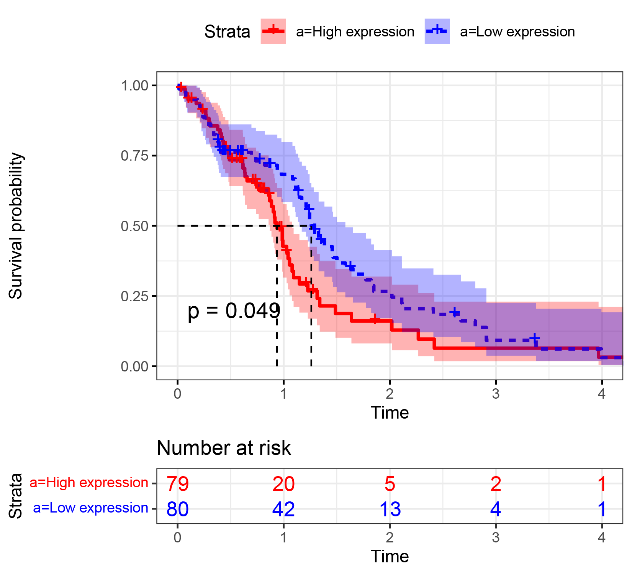


(G) (H)


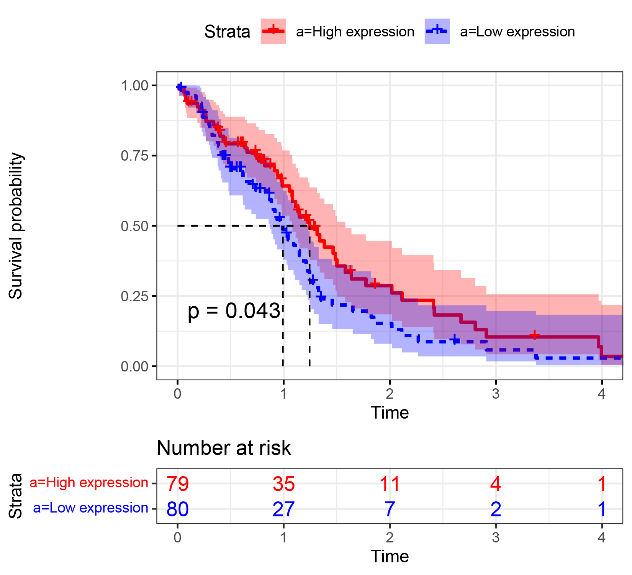

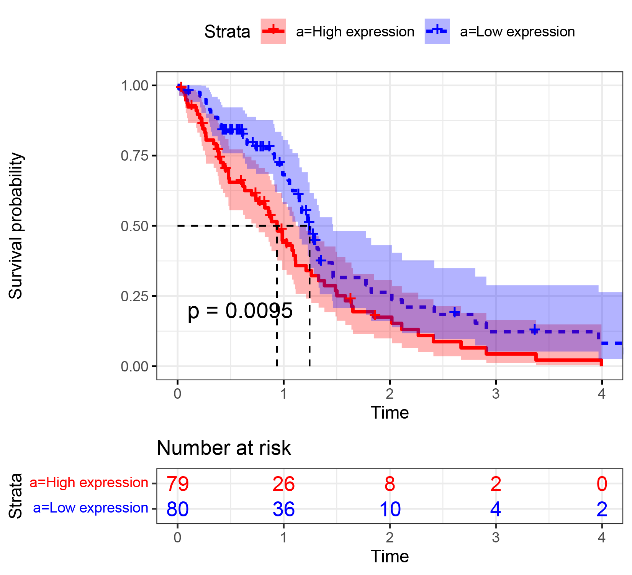


(I) (J)


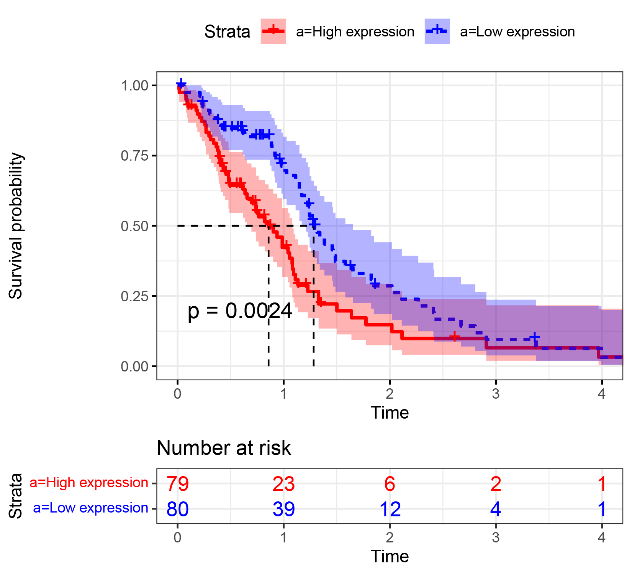

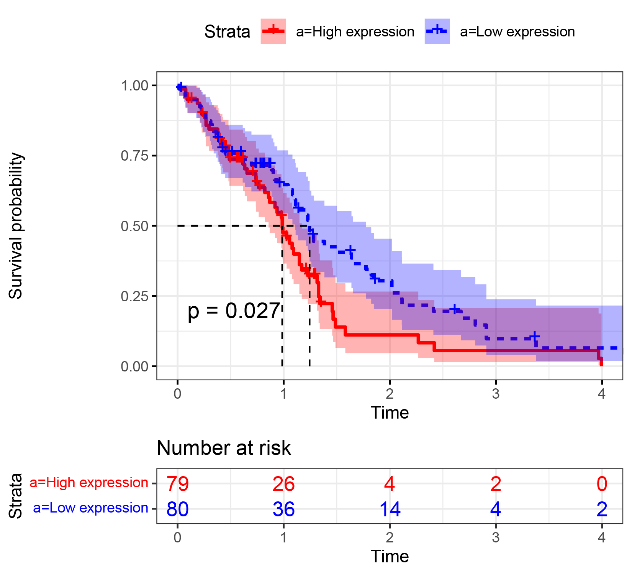


(K) (L)


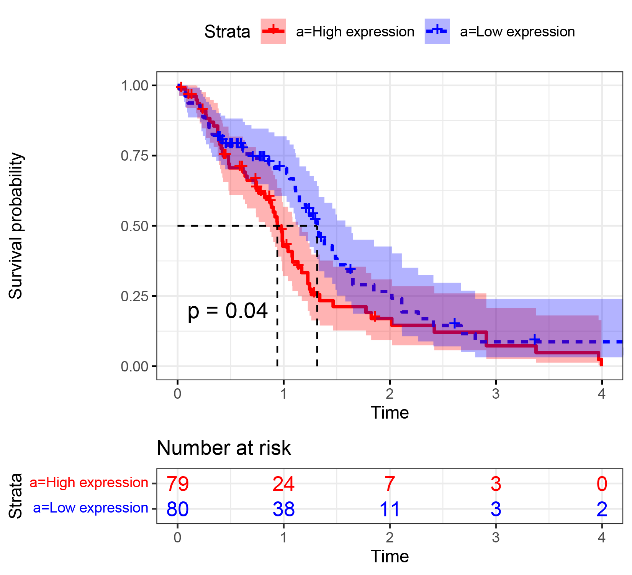

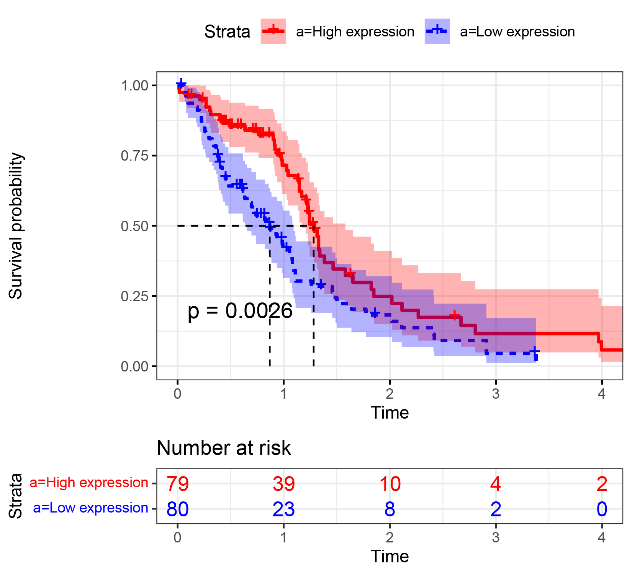


(M) (N)


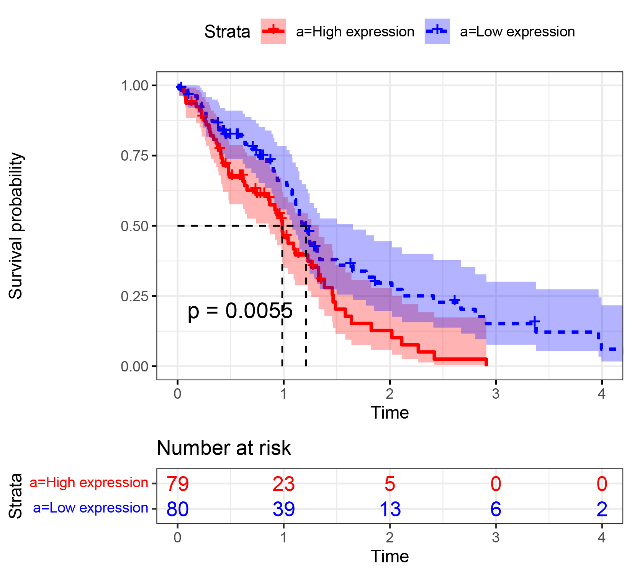


(O)

**Supplementary Figure 1.** Single gene Kaplan-Meier curves of 15 genes obtained by univariate cox regression analysis. (A-O) Single gene Kaplan-Meier curves of CCR2, CD248, FCGR2B, IKBIP, IL32, LILRB2, LYNX1, MMP9, PRELID1P4, RGS14, RGS17P1, RUNX1, TBX15, TCF12, TREM1.

## Supplementary Tables

**Supplementary Table 1.** Expression differences between the tumor and normal groups of 198 DEIRGs.

| Gene | Log2 \| FC \| | *P* value | FDR | Down/up-regulated |
| --- | --- | --- | --- | --- |
| MAL2 | -4.906 | 1.72E-04 | 0.002 | Down |
| KIRREL3-AS3 | -4.764 | 6.55E-05 | 0.002 | Down |
| LY86-AS1 | -4.424 | 2.37E-04 | 0.002 | Down |
| INHBA-AS1 | -4.010 | 2.63E-04 | 0.002 | Down |
| CD22 | -3.817 | 1.26E-03 | 0.004 | Down |
| PYDC1 | -3.768 | 2.07E-04 | 0.002 | Down |
| MAL2-AS1 | -3.709 | 4.76E-06 | 0.002 | Down |
| BNIP3P32 | -3.603 | 5.12E-04 | 0.002 | Down |
| OPRK1 | -3.559 | 2.06E-04 | 0.002 | Down |
| KRT18P39 | -3.514 | 2.07E-03 | 0.005 | Down |
| KRT18P46 | -3.438 | 8.99E-06 | 0.002 | Down |
| CD200R1L | -3.387 | 1.86E-05 | 0.002 | Down |
| DMBT1P1 | -3.377 | 6.26E-05 | 0.002 | Down |
| VIPR1 | -3.375 | 2.64E-04 | 0.002 | Down |
| MAL | -3.369 | 7.79E-04 | 0.003 | Down |
| VIPR1-AS1 | -3.297 | 1.52E-04 | 0.002 | Down |
| KRT16P6 | -3.243 | 5.56E-04 | 0.002 | Down |
| CRHR1 | -3.202 | 2.83E-04 | 0.002 | Down |
| KIRREL3 | -3.129 | 1.44E-04 | 0.002 | Down |
| INSYN2B | -3.002 | 2.29E-04 | 0.002 | Down |
| KRT18P65 | -2.999 | 1.10E-03 | 0.003 | Down |
| SFTPD-AS1 | -2.967 | 1.62E-04 | 0.002 | Down |
| C2CD4C | -2.965 | 2.93E-04 | 0.002 | Down |
| OPRD1 | -2.934 | 5.24E-04 | 0.002 | Down |
| MBP | -2.840 | 5.38E-03 | 0.011 | Down |
| SEMA4D | -2.830 | 2.46E-04 | 0.002 | Down |
| PRLHR | -2.773 | 7.72E-04 | 0.003 | Down |
| LYNX1 | -2.686 | 1.55E-04 | 0.002 | Down |
| CD164L2 | -2.678 | 1.71E-04 | 0.002 | Down |
| GPIHBP1 | -2.659 | 1.67E-03 | 0.004 | Down |
| INSM2 | -2.657 | 1.01E-03 | 0.003 | Down |
| IFNL1 | -2.590 | 1.47E-03 | 0.004 | Down |
| HRH2 | -2.589 | 1.67E-03 | 0.004 | Down |
| BCL2L10 | -2.524 | 9.97E-03 | 0.017 | Down |
| CARTPT | -2.506 | 4.34E-04 | 0.002 | Down |
| DEFB131E | -2.505 | 1.04E-03 | 0.003 | Down |
| KRT17P1 | -2.389 | 5.20E-04 | 0.002 | Down |
| KRT1 | -2.343 | 4.97E-03 | 0.010 | Down |
| C2CD4D | -2.332 | 2.93E-04 | 0.002 | Down |
| BCL2L2 | -2.322 | 1.44E-04 | 0.002 | Down |
| C22orf24 | -2.244 | 3.37E-04 | 0.002 | Down |
| WASF1 | -2.121 | 4.43E-04 | 0.002 | Down |
| CCR9 | -2.092 | 4.74E-04 | 0.002 | Down |
| C2CD2L | -2.009 | 1.49E-04 | 0.002 | Down |
| RGS14 | -2.003 | 2.26E-03 | 0.005 | Down |
| C2orf66 | 2.031 | 4.42E-03 | 0.009 | Up |
| KRT10 | 2.033 | 1.44E-04 | 0.002 | Up |
| IL18 | 2.034 | 1.11E-03 | 0.003 | Up |
| CDC42P4 | 2.046 | 9.42E-03 | 0.017 | Up |
| FTH1P23 | 2.053 | 2.14E-04 | 0.002 | Up |
| IL15RA | 2.055 | 3.37E-04 | 0.002 | Up |
| CTSW | 2.067 | 1.62E-03 | 0.004 | Up |
| RGS18 | 2.094 | 2.06E-03 | 0.005 | Up |
| LY86 | 2.100 | 1.18E-03 | 0.003 | Up |
| ITGB2 | 2.102 | 6.40E-04 | 0.002 | Up |
| CD86 | 2.103 | 6.61E-04 | 0.003 | Up |
| C1QBPP2 | 2.114 | 5.99E-04 | 0.002 | Up |
| TCF12 | 2.117 | 3.25E-04 | 0.002 | Up |
| CDC42P5 | 2.120 | 6.72E-04 | 0.003 | Up |
| CDC42BPG | 2.129 | 2.16E-02 | 0.034 | Up |
| SLA2 | 2.139 | 6.83E-04 | 0.003 | Up |
| CDC42EP5 | 2.149 | 2.19E-03 | 0.005 | Up |
| ELF4 | 2.155 | 2.55E-04 | 0.002 | Up |
| CD96 | 2.157 | 6.00E-03 | 0.011 | Up |
| IL10RB | 2.164 | 1.49E-04 | 0.002 | Up |
| CCR5 | 2.173 | 2.26E-03 | 0.005 | Up |
| CMKLR1 | 2.190 | 7.54E-04 | 0.003 | Up |
| HAMP | 2.197 | 2.79E-02 | 0.042 | Up |
| CD3E | 2.197 | 9.73E-03 | 0.017 | Up |
| TCF7 | 2.206 | 1.85E-04 | 0.002 | Up |
| BST2 | 2.218 | 2.06E-03 | 0.005 | Up |
| IFI6 | 2.218 | 4.55E-03 | 0.009 | Up |
| TRAT1 | 2.223 | 2.93E-02 | 0.044 | Up |
| C2 | 2.242 | 1.34E-03 | 0.004 | Up |
| RGS17P1 | 2.247 | 2.87E-02 | 0.043 | Up |
| IL2RB | 2.267 | 1.83E-03 | 0.005 | Up |
| FTH1P7 | 2.273 | 2.14E-04 | 0.002 | Up |
| IL27 | 2.276 | 2.62E-03 | 0.006 | Up |
| LCK | 2.289 | 2.78E-03 | 0.006 | Up |
| CD28 | 2.298 | 4.95E-03 | 0.010 | Up |
| TCF7L1 | 2.302 | 2.64E-04 | 0.002 | Up |
| LAX1 | 2.308 | 3.32E-03 | 0.007 | Up |
| RPS19 | 2.309 | 1.44E-04 | 0.002 | Up |
| MS4A14 | 2.337 | 7.06E-04 | 0.003 | Up |
| CDK6 | 2.359 | 1.67E-03 | 0.004 | Up |
| TRIM22 | 2.362 | 3.86E-04 | 0.002 | Up |
| SKAP1 | 2.378 | 4.55E-03 | 0.009 | Up |
| CCR4 | 2.389 | 1.09E-02 | 0.019 | Up |
| PRELID1P4 | 2.418 | 5.07E-04 | 0.002 | Up |
| INSM1 | 2.431 | 2.38E-02 | 0.036 | Up |
| ARHGDIB | 2.444 | 1.49E-04 | 0.002 | Up |
| PRELID1P3 | 2.457 | 2.11E-02 | 0.033 | Up |
| CD72 | 2.457 | 3.73E-04 | 0.002 | Up |
| APOBEC3F | 2.462 | 2.14E-04 | 0.002 | Up |
| FTH1P16 | 2.467 | 1.66E-04 | 0.002 | Up |
| IFI16 | 2.471 | 2.73E-04 | 0.002 | Up |
| SCIN | 2.479 | 2.47E-03 | 0.006 | Up |
| FCGR1A | 2.515 | 4.58E-04 | 0.002 | Up |
| IL15 | 2.542 | 5.07E-04 | 0.002 | Up |
| FCGRT | 2.554 | 1.44E-04 | 0.002 | Up |
| CKLF | 2.576 | 1.44E-04 | 0.002 | Up |
| APOBEC3G | 2.578 | 2.93E-04 | 0.002 | Up |
| ACKR4 | 2.590 | 8.09E-03 | 0.015 | Up |
| BST1 | 2.606 | 1.92E-04 | 0.002 | Up |
| NCF4 | 2.614 | 1.79E-04 | 0.002 | Up |
| ERAP2 | 2.615 | 1.74E-02 | 0.028 | Up |
| RUNX1 | 2.624 | 4.28E-04 | 0.002 | Up |
| IL2RG | 2.643 | 2.93E-04 | 0.002 | Up |
| CCL5 | 2.658 | 7.54E-04 | 0.003 | Up |
| BNIP3P27 | 2.689 | 5.66E-03 | 0.011 | Up |
| CST7 | 2.694 | 4.58E-04 | 0.002 | Up |
| CD74 | 2.720 | 2.55E-04 | 0.002 | Up |
| HELLS | 2.736 | 2.64E-04 | 0.002 | Up |
| BCL2A1 | 2.784 | 2.13E-03 | 0.005 | Up |
| CCL26 | 2.800 | 5.53E-03 | 0.011 | Up |
| NCR1 | 2.800 | 1.05E-02 | 0.018 | Up |
| TREM2 | 2.802 | 2.83E-04 | 0.002 | Up |
| C5AR1 | 2.805 | 6.19E-04 | 0.002 | Up |
| FTH1P21 | 2.807 | 4.00E-04 | 0.002 | Up |
| RGS1 | 2.832 | 1.01E-03 | 0.003 | Up |
| CD3D | 2.834 | 2.87E-03 | 0.006 | Up |
| IL7 | 2.852 | 2.21E-04 | 0.002 | Up |
| FCGR3A | 2.857 | 3.86E-04 | 0.002 | Up |
| UBE2NL | 2.869 | 3.86E-04 | 0.002 | Up |
| CTSS | 2.874 | 1.85E-04 | 0.002 | Up |
| CD276 | 2.894 | 1.44E-04 | 0.002 | Up |
| BNIP3P16 | 2.898 | 2.42E-02 | 0.037 | Up |
| TBX1 | 2.909 | 1.70E-02 | 0.027 | Up |
| IL18RAP | 2.921 | 2.66E-03 | 0.006 | Up |
| C2orf48 | 2.927 | 5.76E-03 | 0.011 | Up |
| CTSC | 2.940 | 1.55E-04 | 0.002 | Up |
| FTH1P5 | 2.943 | 1.60E-04 | 0.002 | Up |
| CNR2 | 2.952 | 8.57E-03 | 0.015 | Up |
| AIM2 | 2.958 | 1.72E-03 | 0.005 | Up |
| LILRB2 | 2.971 | 3.61E-04 | 0.002 | Up |
| BCL2L12 | 2.972 | 1.44E-04 | 0.002 | Up |
| DPP4 | 3.013 | 2.62E-03 | 0.006 | Up |
| CCL25 | 3.026 | 1.88E-03 | 0.005 | Up |
| FTH1P8 | 3.027 | 1.44E-04 | 0.002 | Up |
| IL17B | 3.034 | 1.15E-03 | 0.003 | Up |
| FTH1P12 | 3.080 | 1.55E-04 | 0.002 | Up |
| IL32 | 3.097 | 3.61E-04 | 0.002 | Up |
| FTH1P2 | 3.148 | 1.44E-04 | 0.002 | Up |
| IL10 | 3.156 | 7.79E-04 | 0.003 | Up |
| RGS13 | 3.156 | 5.08E-03 | 0.010 | Up |
| FTH1P1 | 3.162 | 1.44E-04 | 0.002 | Up |
| CD1D | 3.169 | 2.29E-04 | 0.002 | Up |
| TNFSF13B | 3.188 | 2.29E-04 | 0.002 | Up |
| RPS19P3 | 3.193 | 1.44E-04 | 0.002 | Up |
| CXCR4 | 3.206 | 1.79E-04 | 0.002 | Up |
| CD2 | 3.207 | 1.18E-03 | 0.003 | Up |
| CCR2 | 3.224 | 3.13E-03 | 0.007 | Up |
| IKBIP | 3.226 | 1.44E-04 | 0.002 | Up |
| GZMA | 3.280 | 1.47E-03 | 0.004 | Up |
| WASF4P | 3.286 | 2.14E-04 | 0.002 | Up |
| MAP3K7CL | 3.302 | 2.46E-04 | 0.002 | Up |
| IL4 | 3.316 | 4.64E-03 | 0.010 | Up |
| TLR8 | 3.333 | 7.06E-04 | 0.003 | Up |
| FCGR3B | 3.354 | 9.77E-04 | 0.003 | Up |
| GBP2 | 3.367 | 1.85E-04 | 0.002 | Up |
| CD44 | 3.416 | 2.37E-04 | 0.002 | Up |
| CDC42P6 | 3.448 | 1.44E-04 | 0.002 | Up |
| IL24 | 3.453 | 3.25E-04 | 0.002 | Up |
| FTH1P11 | 3.466 | 1.44E-04 | 0.002 | Up |
| SIT1 | 3.527 | 4.00E-04 | 0.002 | Up |
| FTH1P4 | 3.556 | 1.44E-04 | 0.002 | Up |
| CD248 | 3.649 | 1.72E-04 | 0.002 | Up |
| CCL20 | 3.679 | 1.32E-02 | 0.022 | Up |
| UBE2NP1 | 3.742 | 4.65E-04 | 0.002 | Up |
| TGFB1I1 | 3.773 | 1.44E-04 | 0.002 | Up |
| TBX15 | 3.834 | 1.60E-04 | 0.002 | Up |
| CD48 | 3.867 | 1.85E-04 | 0.002 | Up |
| CD24P4 | 3.884 | 5.67E-03 | 0.011 | Up |
| CXCR2 | 3.903 | 2.29E-04 | 0.002 | Up |
| CXCR2P1 | 4.007 | 1.83E-03 | 0.005 | Up |
| PDCD1LG2 | 4.065 | 1.72E-04 | 0.002 | Up |
| CCL24 | 4.231 | 1.13E-02 | 0.019 | Up |
| FTH1P10 | 4.308 | 1.44E-04 | 0.002 | Up |
| GPR65 | 4.325 | 1.44E-04 | 0.002 | Up |
| CCL23 | 4.350 | 5.19E-03 | 0.010 | Up |
| MNX1-AS2 | 4.391 | 7.86E-03 | 0.014 | Up |
| CD207 | 4.466 | 4.74E-04 | 0.002 | Up |
| C21orf62 | 4.477 | 2.55E-04 | 0.002 | Up |
| CDC42P2 | 4.504 | 7.25E-04 | 0.003 | Up |
| FCGR2B | 4.761 | 2.37E-04 | 0.002 | Up |
| TREM1 | 4.986 | 3.14E-04 | 0.002 | Up |
| KRT17P4 | 5.004 | 2.88E-03 | 0.006 | Up |
| RPS19P7 | 5.123 | 1.44E-04 | 0.002 | Up |
| CD70 | 5.456 | 8.32E-04 | 0.003 | Up |
| RPS19P1 | 5.552 | 1.44E-04 | 0.002 | Up |
| IL1R2 | 5.738 | 2.14E-04 | 0.002 | Up |
| IL2RA | 6.144 | 3.86E-04 | 0.002 | Up |
| CCL18 | 7.473 | 2.40E-03 | 0.006 | Up |
| CXCL13 | 7.480 | 7.92E-04 | 0.003 | Up |
| MMP9 | 7.538 | 1.49E-04 | 0.002 | Up |
| MNX1 | 8.213 | 4.13E-04 | 0.002 | Up |
| LTF | 8.699 | 1.44E-04 | 0.002 | Up |
| SAA1 | 9.130 | 1.66E-04 | 0.002 | Up |

**Supplementary Table 2.** Functional enrichment analysis of GO terms for 198 DEIRGs.

| ONTOLOGY | ID | Description | GeneRatio | Bg  Ratio | *P* value |
| --- | --- | --- | --- | --- | --- |
| BP | GO:0042110 | T cell activation | 33/144 | 474/18862 | 1.49E-22 |
| BP | GO:0050863 | regulation of T cell activation | 28/144 | 327/18862 | 1.36E-21 |
| BP | GO:1903037 | regulation of leukocyte cell-cell adhesion | 28/144 | 330/18862 | 1.74E-21 |
| BP | GO:0007159 | leukocyte cell-cell adhesion | 29/144 | 366/18862 | 2.10E-21 |
| BP | GO:0060326 | cell chemotaxis | 27/144 | 306/18862 | 3.50E-21 |
| BP | GO:0030595 | leukocyte chemotaxis | 24/144 | 226/18862 | 8.45E-21 |
| BP | GO:0022407 | regulation of cell-cell adhesion | 30/144 | 437/18862 | 2.41E-20 |
| BP | GO:0022409 | positive regulation of cell-cell adhesion | 25/144 | 276/18862 | 6.21E-20 |
| BP | GO:0070661 | leukocyte proliferation | 26/144 | 312/18862 | 8.65E-20 |
| BP | GO:0070098 | chemokine-mediated signaling pathway | 17/144 | 88/18862 | 1.49E-19 |
| CC | GO:0009897 | external side of plasma membrane | 28/150 | 402/19520 | 4.58E-19 |
| CC | GO:0001772 | immunological synapse | 5/150 | 41/19520 | 1.50E-05 |
| CC | GO:0045121 | membrane raft | 11/150 | 323/19520 | 4.15E-05 |
| CC | GO:0098857 | membrane microdomain | 11/150 | 323/19520 | 4.15E-05 |
| CC | GO:0098802 | plasma membrane signaling receptor complex | 10/150 | 318/19520 | 1.79E-04 |
| CC | GO:0031225 | anchored component of membrane | 7/150 | 170/19520 | 3.48E-04 |
| MF | GO:0140375 | immune receptor activity | 20/146 | 136/18337 | 5.59E-20 |
| MF | GO:0004896 | cytokine receptor activity | 17/146 | 97/18337 | 1.76E-18 |
| MF | GO:0005125 | cytokine activity | 21/146 | 235/18337 | 2.21E-16 |
| MF | GO:0048018 | receptor ligand activity | 25/146 | 486/18337 | 9.99E-14 |
| MF | GO:0030546 | signaling receptor activator activity | 25/146 | 492/18337 | 1.32E-13 |
| MF | GO:0005126 | cytokine receptor binding | 19/146 | 270/18337 | 4.99E-13 |
| MF | GO:0001637 | G protein-coupled chemoattractant receptor activity | 8/146 | 26/18337 | 1.84E-11 |
| MF | GO:0004950 | chemokine receptor activity | 8/146 | 26/18337 | 1.84E-11 |
| MF | GO:0048020 | CCR chemokine receptor binding | 9/146 | 46/18337 | 8.60E-11 |
| MF | GO:0008009 | chemokine activity | 9/146 | 49/18337 | 1.57E-10 |

Abbreviations: GO, gene ontology; DEIRGs, differently expressed immune-related genes.

Notes:

BgRatio = M/N, N represents the entire gene set, and M contains genes for the specific item in the entire gene set.

GeneRatio = m/n, n represents the overlap between a specific item in the input gene set and the entire gene set, and m represents the overlap between the input gene set and the entire gene set.

**Supplementary Table 3.** Functional enrichment analysis of KEGG terms for 198 DEIRGs.

| ID | Description | Gene  Ratio | Bg  Ratio | *P* value |
| --- | --- | --- | --- | --- |
| hsa04060 | Cytokine-cytokine receptor interaction | 34/99 | 295/8095 | 8.80E-25 |
| hsa04061 | Viral protein interaction with cytokine and cytokine receptor | 23/99 | 100/8095 | 8.14E-24 |
| hsa04672 | Intestinal immune network for IgA production | 10/99 | 49/8095 | 2.61E-10 |
| hsa04640 | Hematopoietic cell lineage | 11/99 | 99/8095 | 2.70E-08 |
| hsa04062 | Chemokine signaling pathway | 14/99 | 192/8095 | 7.21E-08 |
| hsa05150 | Staphylococcus aureus infection | 9/99 | 96/8095 | 2.28E-06 |
| hsa04630 | JAK-STAT signaling pathway | 11/99 | 162/8095 | 4.05E-06 |
| hsa04659 | Th17 cell differentiation | 9/99 | 108/8095 | 6.10E-06 |
| hsa05323 | Rheumatoid arthritis | 8/99 | 93/8095 | 1.63E-05 |
| hsa05140 | Leishmaniasis | 7/99 | 77/8095 | 3.95E-05 |
| hsa05152 | Tuberculosis | 10/99 | 180/8095 | 6.47E-05 |
| hsa04658 | Th1 and Th2 cell differentiation | 7/99 | 92/8095 | 1.24E-04 |
| hsa04380 | Osteoclast differentiation | 8/99 | 128/8095 | 1.61E-04 |
| hsa05162 | Measles | 8/99 | 139/8095 | 2.84E-04 |
| hsa05166 | Human T-cell leukemia virus 1 infection | 10/99 | 222/8095 | 3.62E-04 |
| hsa05330 | Allograft rejection | 4/99 | 38/8095 | 1.13E-03 |
| hsa05340 | Primary immunodeficiency | 4/99 | 38/8095 | 1.13E-03 |
| hsa05321 | Inflammatory bowel disease | 5/99 | 65/8095 | 1.14E-03 |
| hsa04666 | Fc gamma R-mediated phagocytosis | 6/99 | 97/8095 | 1.16E-03 |
| hsa05221 | Acute myeloid leukemia | 5/99 | 67/8095 | 1.31E-03 |
| hsa05322 | Systemic lupus erythematosus | 7/99 | 136/8095 | 1.33E-03 |
| hsa04660 | T cell receptor signaling pathway | 6/99 | 104/8095 | 1.66E-03 |
| hsa04514 | Cell adhesion molecules | 7/99 | 149/8095 | 2.25E-03 |
| hsa04145 | Phagosome | 7/99 | 152/8095 | 2.52E-03 |
| hsa05320 | Autoimmune thyroid disease | 4/99 | 53/8095 | 3.90E-03 |
| hsa04650 | Natural killer cell mediated cytotoxicity | 6/99 | 131/8095 | 5.25E-03 |
| hsa04613 | Neutrophil extracellular trap formation | 7/99 | 190/8095 | 8.50E-03 |
| hsa05202 | Transcriptional misregulation in cancer | 7/99 | 192/8095 | 8.98E-03 |
| hsa05167 | Kaposi sarcoma-associated herpesvirus infection | 7/99 | 194/8095 | 9.48E-03 |

Abbreviations: KEGG, kyoto encyclopedia of genes and genomes.

**Supplementary Table 4.** Protein node degrees of five marker genes and their related genes.

| Node | Identifier | Node Degree |
| --- | --- | --- |
| CCL20 | 9606.ENSP00000351671 | 4 |
| CCL5 | 9606.ENSP00000474412 | 10 |
| CCR2 | 9606.ENSP00000292301 | 9 |
| CD44 | 9606.ENSP00000398632 | 8 |
| FCGR2B | 9606.ENSP00000351497 | 7 |
| IL10 | 9606.ENSP00000412237 | 10 |
| IL2RA | 9606.ENSP00000369293 | 5 |
| ITGB2 | 9606.ENSP00000380948 | 10 |
| MMP9 | 9606.ENSP00000361405 | 8 |
| RGS1 | 9606.ENSP00000356429 | 4 |
| RGS13 | 9606.ENSP00000442837 | 3 |
| RGS14 | 9606.ENSP00000386229 | 4 |
| RGS18 | 9606.ENSP00000356430 | 5 |
| RUNX1 | 9606.ENSP00000300305 | 2 |
| SAA1 | 9606.ENSP00000384906 | 4 |
| TCF12 | 9606.ENSP00000388940 | 1 |
| TREM1 | 9606.ENSP00000244709 | 6 |
| CCL20 | 9606.ENSP00000351671 | 4 |
| CCL5 | 9606.ENSP00000474412 | 10 |
| CCR2 | 9606.ENSP00000292301 | 9 |
| CD44 | 9606.ENSP00000398632 | 8 |
| FCGR2B | 9606.ENSP00000351497 | 7 |
| IL10 | 9606.ENSP00000412237 | 10 |
| IL2RA | 9606.ENSP00000369293 | 5 |
| ITGB2 | 9606.ENSP00000380948 | 10 |
| LYNX1 | 9606.ENSP00000479586 | 0 |
| MMP9 | 9606.ENSP00000361405 | 8 |
| RGS1 | 9606.ENSP00000356429 | 4 |
